# Modeling and optimization of surface modification process of ultrafiltration membranes by guanidine-based deep eutectic solvent

**DOI:** 10.1016/j.heliyon.2024.e41432

**Published:** 2024-12-21

**Authors:** Foad Gholami, Azar Asadi, Mina Dolatshah, Safoora Nazari

**Affiliations:** a Environmental Group, Energy Department, Materials and Energy Research Centre, Alborz, Iran; b Department of Applied Chemistry, Faculty of Gas and Petroleum, Yasouj University, Gachsaran, 75918-74831, Iran; c Department of Applied Chemistry, Faculty of Chemistry, Razi University, 67144-14971, Kermanshah, Iran

**Keywords:** Polyetherimide (PEI) membrane, Deep eutectic solvent (DES), Surface-modified membrane, Modeling and optimization, Antibiotic separation

## Abstract

Low performance and the high fouling tendency of Polyetherimide (PEI) membranes prevent their widespread commercial utility. In this study, we utilized a deep eutectic solvent (DES) as a versatile agent for surface modification of the PEI membrane using a simple and sustainable method. To attain an efficient PEI membrane, modeling and optimization of the modification condition were conducted via response surface methodology (RSM). The effects of two effective variables, including the choline/guanidine (Ch/Gu) ratio (0.5–2) and modification time (12–48 h), were evaluated on four responses, i.e., pure water flux (PWF), flux recovery ratio (FRR), irreversible fouling ratio (R_ir_), and total resistance (R_t_). The structural and chemical characteristics and filtration performance of the fabricated membranes were evaluated. The optimum condition was obtained at a 0.8 Ch/Gu ratio and 30 h of modification time to reach the best performance of the DES-PEI membrane. The industrial application of optimally modified DES/PEI membrane was investigated by filtering penicillin and cephalexin antibiotics. The rejection data showed higher performance of the modified membrane (>95 %) compared to the bare membrane (∼63 %). Furthermore, the long-term filtration evaluation in dead-end and cross-flow setups indicated stable flux and rejection of the DES/PEI-modified membrane. The DES, including Ch/Gu, can be viewed as an agent to enhance both PWF and antifouling properties while broadening membrane applications in separating antibiotics for PEI membranes through an eco-friendly and cost-effective method.

## Highlights

• Polyetherimide (PEI) membrane was surface-modified by anchoring DES.
• DES was synthesized by incorporating guanidine (Gu) and choline chloride (Ch).
• Two variables, including (Ch/Gu) ratio and modification time, were investigated.
• The membrane fabricating conditions were designed using Design Expert software.
• The long-term penicillin rejection by the optimal coated membrane was achieved.

## Graphical abstract

**Figure:**
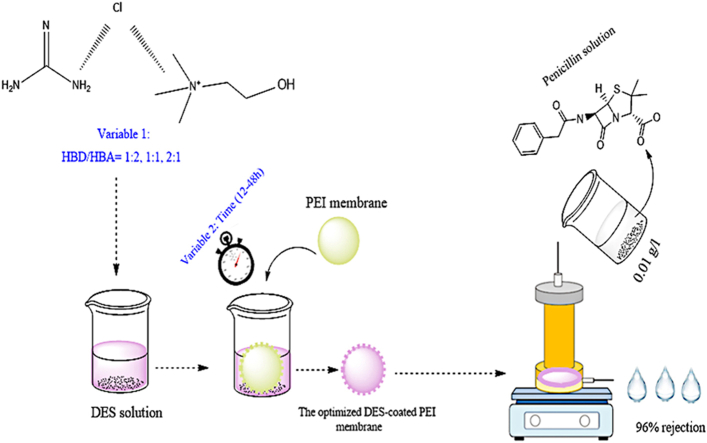

## 1 Introduction

Polyetherimide (PEI) membranes are commonly used for numerous separation processes including gas separation and water filtration [1,2]. PEI membranes have several benefits like their physical and mechanical properties. Nowadays, composite PEI membranes are developed for commercial filtration processes. However, the surface roughness and polymeric nature of PEI membranes make them highly susceptible to fouling. Furthermore, the surface charge is one of the substantial issues that increases membrane fouling via stimulation of foulants' functional groups. Therefore, amelioration of roughness and surface charge is necessary to overcome PEI membrane fouling [3]. Recently, a comprehensive review evaluated the different compounds that have been used for membrane surface modification to control fouling, including nanomaterials, polyelectrolytes, inorganic salts, and polymers (neutral, ionic, and zwitterionic). However, the toxic and expensive materials and the volatile organic solvents required make it not eco-friendly [4,^5^,6].

In the last few years, research in green chemicals has come to explore deep eutectic solvents (DESs) as promising cheap and eco-friendly alternatives to ionic liquids (ILs) [7], 8], 9]. DESs and ILs compared to conventional solvents have some advantages including a relatively wide liquid-range, low vapor pressure, and non-flammability properties [10]. Furthermore, DESs have gained enormous attention owing to their user-friendly properties such as non-toxicity, good biodegradability, high dissolution abilities, extensive tunability, chemical and thermal stability, multitasking applicability, non-flammability, low cost, low melting point, high dissolution ability, and the potential for scalability [11,12]. Generally, DESs are synthesized from H-bond acceptors (HBA, the complexation of quaternary ammonium salts) and H-bond donors (HBD, for instance, amides, carboxylic acids, and alcohols) [13]. Choline chloride (Ch) is a preferred and promising quaternary ammonium salt owing to its biocompatibility and high ability to form an intermolecular H-bond [14,15]. Therefore, DESs have received tremendous attention in fields of ionothermal synthesis [16], metal electrodeposition [17], lithium ion batteries [18], organic synthesis [19], and dye-sensitized solar cells [20]. It is well-known that ILs have a high ability to dissolve several polymers that are not soluble in conventional solvents [21]. It can be inferred that the nature of DESs can also potentially develop considerable solubility and compatibility with polymers in membrane preparation. Compared to ILs, DESs are a more appropriate solvent in industry due to their low production cost and relative ease of preparation. Recent research on DESs has resulted in the expansion of their applications, especially in membrane technology [22,23]. So far, most DES applications in the field of membrane technology have been limited to being entrapped in the polymer matrix for applications such as pervaporation, gas separation, and solvent extraction [24].

However, to our knowledge, there is no report on the modeling and optimization of modification PEI membranes using DES based on an accurate evaluation of their physicochemical and performance properties. Herein, for the first time, Choline/Guanidine (Ch/Gu) DES was used to modify the PEI membrane. The effects of two functional variables, including the Choline/Guanidine ratio and the modification time, on the four responses of PWF, FRR, R_ir_, and R_t_, were assessed. To achieve this aim, a response methodology surface (RSM)-based central composite design (CCD) was used for experimental design using Design Expert software. The effect of DESs treatment on membrane structure, morphology, properties, permeability, and antifouling properties is discussed in detail.

## 2 Experimental

### 2.1 Materials

Polyetherimide (PEI, Mw: 592.61 g/mol) and Poly (Polyvinyl pyrrolidone) (PVP, Mw = 25,000 g/mol) were procured from Sigma-Alderich-US. Choline chloride (ChCl, 99.9 %) and Guanidine (Gu, 99.0 %) were purchased from Merck Co., Germany. Also, N-Methyl-2-pyrrolidone (NMP) was supplied by Merck, Germany. Penicillin and cephalexin were purchased from Pars Pharmaceutical Co., Iran. Deionized (DI) water was used throughout the experiments.

### 2.2 Preparation of deep eutectic solvent (DES)

The DESs were prepared via a typical reaction, as described in the earlier literature [25]. Generally, accroding to Table 1, different molar ratios of choline chloride (a hydrogen bond acceptor) and guanidine (hydrogen bond donor) were mixed in 100-mL glass containers, followed by stirring at 80 °C for 2 h until clear homogeneous liquids were reached.

**Table 1:** Experimental range and levels of the variables.

**Variables**	**Range and levels**		
	**−1**	**0**	**+1**
A: Choline/Guanidine ratio	0.5	1.25	2
B: Modification time (h)	12	30	48

### 2.3 PEI membranes preparation

The bare ultrafiltration (UF) membrane was prepared via the non-solvent induced phase inversion method based on the literature. Briefly, PEI polymer (16.5 wt%) and PVP pore former (4 wt%) were dissolved in NMP solvent under continuous stirring (500 rpm) at 40 °C for 24 h. The casting solution was ultrasonically degassed for 15 min. The prepared solution was spattered on a glass support using a casting knife with a 150 μm thickness and immediately submerged in a coagulation bath (DI water, non–solvent) overnight at room temperature. The pristine membrane (M_1_) was completely dried by being interposed between two sheets of filter paper.

### 2.4 DES treatment on PEI membrane

To achieve a modified PEI membrane with high performance via DES, two important factors, including Choline/Guanidine (Ch/Gu) ratio and modification time, were evaluated (see Table 1). To this end, CCD-based RSM via Design Expert software (version 11.1.1) was utilized to investigate the influence of selected numerical variables at three levels (2k + (2k+1) +5), where k is the variable's number. The experimental range of variables was chosen based on earlier research: 0.5–2 Ch/Gu ratio and 12–48 h for modification time [27]. Table 2 tabulates the experimental range and independent variable levels. Further information on the exact experimental design using CCD is accessible in the literature [26].

**Table 2:** Experimental conditions and the obtained results.

**Membrane**	Variables		Responses			
	A:Ch/Gu Ratio	B:Time (h)	PWF (L/m^2^.h)	FRR (%)	R_ir_ (%)	R_t_ (m^−1^)
M_2_	1.25	12	5.97	84.62	15.37	4.23e+13
M_3_	0.5	12	9.35	88.32	11.67	2.97e+13
M_4_	2	12	7.38	81.35	18.65	3.85e+13
M_5_	1.25	30	11.59	92.32	7.68	2.27e+13
M_5_	1.25	30	10.84	92.69	7.94	2.27e+13
M_5_	1.25	30	11.93	88.41	7.59	2.34e+13
M_5_	1.25	30	11.84	89.63	8.37	2.21e+13
M_5_	1.25	30	10.3	92.15	8.38	2.25e+13
M_6_	0.5	30	13.88	94.25	5.75	1.97e+13
M_7_	2	30	8.27	91.24	8.75	3.17e+13
M_8_	1.25	48	9.53	81.36	18.63	3.07e+13
M_9_	0.5	48	11.88	84.32	15.67	2.56e+13
M_10_	2	48	4.84	79.21	20.78	4.17e+13

According to the designed conditions, the circularly cut pristine PEI membranes (d = 5 cm) were soaked by DES with different Ch/Gu ratios and modification times. After modification, the coated PEI membranes were washed with DI water and dried. The treated membranes were named M_2_-M_10_, respectively (Table 2).

The membranes' performance was assessed by measuring four responses, i.e., pure water flux (PWF, L/m^2^.h), flux recovery ratio (FRR, %), irreversible fouling ratio (R_ir_, %), and total resistance (R_t_, m^−1^).

### 2.5 Membranes characterization

Attenuated total reflection infrared spectroscopy (ATR-IR, Bruker TENSOR27 spectrometer) over the wavelength range of 400–4000 cm^−1^ was utilized to identify changes in the functional groups on the surface of pristine and DES-treated PEI membranes. The zeta potential of the fabricated membranes was measured via a Physica EKA Electro Kinetic instrument (Anton Paar, Austria) at various pH values of 3–13 to indicate the surface load significance in the membrane separation approach. A scanning electron microscope (SEM, Tescan MIRA3) was employed to evaluate the top surface and cross-section morphologies of the pristine membrane and DES-treated PEI membranes. Before cross-section analysis, the membranes were fractured at liquid nitrogen temperature. Also, all membrane samples were sputter-coated with a thin coating of gold under vacuum to prepare for SEM analysis at 20 kV. The topographical profiles of the membranes' surfaces were assessed by an atomic force microscope (AFM, Nanosurf® scanning probe-optical microscope, Switzerland). The AFM images were obtained by scanning a small surface area of membranes (3 μm × 3 μm). Then, the imaging data were further analyzed via Nanosurf® Mobile software to estimate roughness indices. The results were represented in terms of the surface roughness parameters, including mean roughness (S_a_), the root mean square of the Z data (S_q_), and the mean difference between the highest peaks and lowest valleys (S_z_). In order to assess the water contact angle (WCA), the sessile drop goniometer method was used. A water droplet of 5 μL was put on the top surface of the membrane specimen. Ten random sites were selected for each membrane sample, and average quantities were represented to alleviate measurement error. The Young-Dupre equation was applied to determine the contribution of membrane surface free energy (-ΔGs) to its antifouling properties. The Young-Dupre for a water environment is represented by the following Equation:

$$-\Delta \text{Gs}=\left(1+\cos\;\theta \right)\gamma_{L}$$

where -ΔGs the free energy of interaction at the liquid-surface interface, θ is the empirically determined contact angle, and γ_L_ the liquid's surface tension at ambient temperature (72.8 mJ/m^2^).

### 2.6 Evaluation of membrane performance

All experiments were conducted in a house-in dead-end stirred filtration setup with an effective area of 19.625 cm^2^ and a processing capacity of 250 mL under nitrogen gas to provide the requisite pressure. In the first stage of the filtration studies, the membranes were compressed at 5 bar for 60 min to stabilize the flow, and then the pure water flux (PWF) was measured at a pressure of 3 bar. After collecting and weighing the permeate samples over a set length of time, the PWF (J_W.1_, L/m^2^.h) of the membranes was calculated using the gravimetric method according to the following formula:

$$j_{w.1}=\frac{M}{A\Delta t}$$

where PWF, via an effective membrane surface area (A), was calculated as the weight of collected permeate (M) during a certain time period (Δt).

To assess the antifouling properties of the DES-PEI membranes, a milk powder solution (1000 ppm) was used as an organic foulant representative. After measuring the water flux rate for the first run (J_w.1_), the permeate flux of the milk powder solution was evaluated for 2.5 h at 3 bar (J_p_). The fouled membrane was then taken out, washed with distilled water, and reused under experimental conditions. The cleaned membrane's PWF for the second run (J_w.2_) was measured and monitored. The flux recovery ratio (FRR, %) was determined to evaluate the fouling propensity using the equation below:

$$\text{FRR}=\left(\frac{J_{w.2}}{J_{w.1}}\right)\times 100$$

where J_w.1_ and J_w.2_ represent the initial PWF and the recovered permeate flux after filtering the milk powder solution, respectively.

The fouling resistance of DES-PEI membranes was characterized using the total resistance (R_t_) and irreversible fouling (R_ir_) ratios via the following equations to identify the fouling process:

$$R_{t}\left(\%\right)=\left(1-\frac{J_{p}}{J_{w.1}}\right)\times 100$$

$$R_{ir}\left(\%\right)=\left(\frac{J_{w.1}-J_{w.2}}{J_{w.1}}\right)\times 100$$

All experiments for each membrane were repeated three times, and the average of the data was provided in Table 2 as responses of the filtration process.

In the second stage, the efficiency of the bare and optimally modified membranes for rejection of two antibiotics, penicillin and cephalexin, was assessed at an initial concentration of 10 mg/L and pH of 7. The feed (C_f_) and permeate (C_p_) concentrations of penicillin and cephalexin with a maximum absorption wavelength of 237 nm and 258 nm, respectively, were measured by a UV–vis spectrophotometer (Hach DR 5000), and the rejection was calculated using the following equation:

$$R\;\left(\%\right)=\left(1-\frac{C_{p}}{C_{f}}\right)\times 100$$

In order to conduct long-term filtration studies, 0.01 g/L penicillin antibiotic solution at pH = 7 and 4 bar was filtered in both the dead-end and cross-flow set-ups from the bare and optimally modified membranes. A long-term 300-min filtration evaluation was carried out in the cross-flow system, using a flow rate of 120 L/h. The assessment included a cell with a membrane area of 5 cm^2^.

## 3 Results and discussion

### 3.1 Membrane characterization

#### 3.1.1 Membrane surface chemistry

**Fig. 1:**
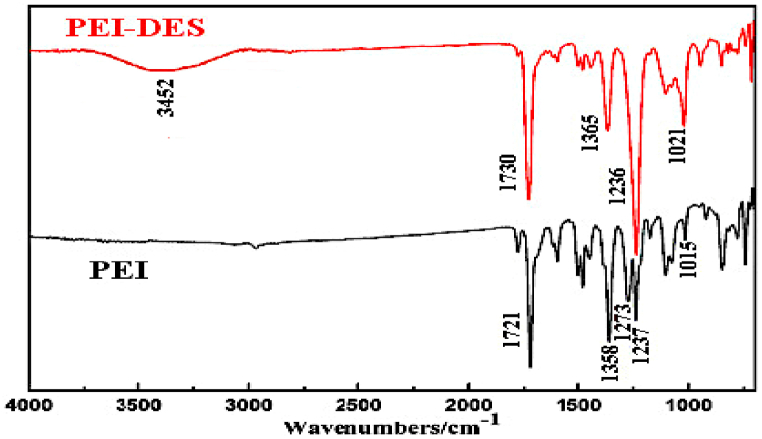
Surface ATR-FTIR spectra of the PEI and DES-PEI membranes.

**Fig. 2:**
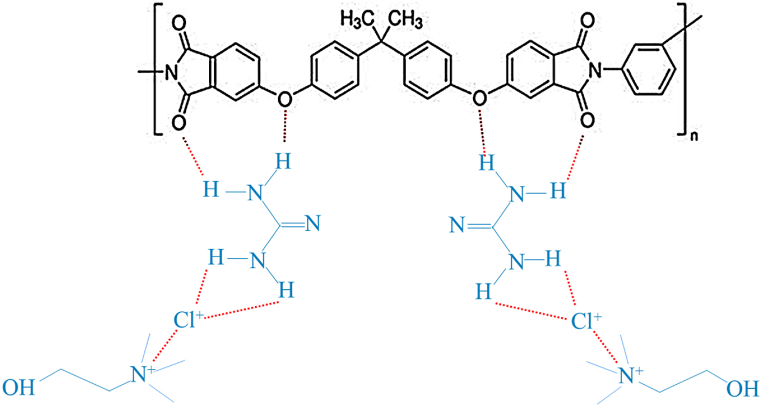
Chemical structure of PEI and active layer.

The characteristic bands of the PEI and DES-PEI membranes were indicated in the ATR-FTIR spectra in Fig. 1. The ATR-IR spectrum of pristine PEI reveals the characteristic peaks at 1015, 1273–1237, 1358, 1721, and 1499-1446 cm^−1^, which were ascribed to C-O-C symmetric and asymmetric stretching, C-H bending and stretching, C=O stretching, and C=C stretching of the PEI backbone, respectively. Most of the aforementioned PEI polymer signature peaks could be observed in DES-PEI spectrum. However, the C-O-C asymmetric stretching vibration peak was converted into a strong single peak at 1236 cm^−1^ in the DES-PEI spectrum. Also, C-H, C=O, and C-O-C symmetric stretching vibrations shifted from 1358 to 1365 cm^−1^, 1721–1736 cm^−1^, and 1015–1021 cm^−1^, respectively. It was also observed that the DES-PEI membrane had higher relative peak area ratios for C-H bending, C=O stretching, and C-O-C asymmetric and symmetric stretching vibrations compared to the pristine PEI. These confirmed the presence of the DES layer on the PES support. Moreover, a broad peak between 3000 and 3500 cm^−1^ could be observed in PEI-DES, ascribed to hydrogen bonding between choline chloride and guanidine in DES (as revealed in Fig. 2) which could be considered as another sign of the presence of DES on PEI membrane.

Fig. 2 portrays the interaction between the PEI membrane surface and the DES (choline chloride and guanidine) after modification which is primarily based on hydrogen bonding and electrostatic interactions. The nitrogen atoms in guanidine form hydrogen bonds with the hydrogen atoms on the PEI membrane, while the hydroxyl (-OH) group in choline contributes additional hydrogen bonding. Additionally, the chloride ion (Cl⁻) in choline chloride interacts electrostatically with positively charged regions on the membrane surface. These combined interactions lead to a stable DES coating on the PEI membrane, enhancing some properties such as hydrophilicity, fouling resistance, and PWF.

**Fig. 3:**
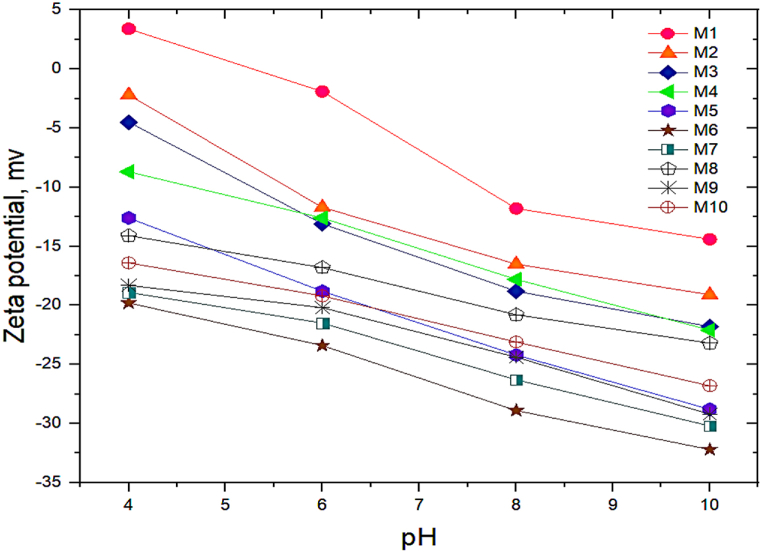
Zeta potential of the prepared membranes versus pH.

Besides, the membrane's surface charge controls the mass transport of charged species and subsequently fouling behavior by influencing the electrostatic attraction or repulsion of charged species by the membranes. Accordingly, the surface charge of pristine PEI and DES-PEI membranes was evaluated by zeta potential analysis. The zeta potential data for M_1_ to M_10_ membranes at pH 4 to 10 have been provided in Fig. 3. The pristine PEI membrane indicated the point of isoelectricity (PIC) at pH 5.5. However, by decreasing pH (pH 4), protonation of the amine functional groups caused the positively charged membrane surfaces. An increase in pH up to 10 led to a negative membrane surface charge owing to the deprotonation of amine and carboxylic acid groups. Similar observations have been reported for the PEI membranes in the literature as well [27]. PIC of the modified membranes shifted to lower pH so that the zeta potential of modified DES-PEI membranes was negative at all tested pH values (4–10). These appreciable changes could be related to the presence of amine groups on the membrane surface and H-bonding interactions between the DES and PEI membranes during the modification treatment, as depicted in Fig. 2. It's worth mentioning that M_6_ had the highest negative zeta potential values in the applied pH range. M_6_ was modified at the lowest Ch/Gu ratio (0.5), corroborating the effective modification to provide negative functional groups on the surface, however, at higher Ch/Gu ratio, coagulation on the surface could be occurred which reduces free fictional groups on the surface.

#### 3.1.2 Membrane morphology and topography

**Fig. 4:**
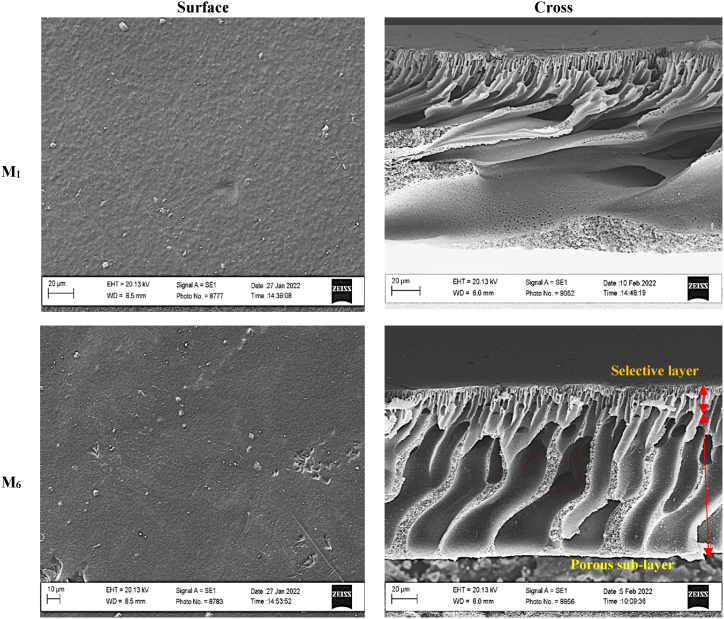
Surface and cross-section SEM images of the bare membrane (M_1_) and optimum DES-PEI membrane (M_6_).

Since the membrane morphology influences its permeability and antifouling properties, the surface and cross-section morphologies of the pristine and DES-PEI membranes were evaluated via SEM images in Supplementary 1. As evidenced in the cross-section of the fabricated membranes, all membranes include the same support PEI matrix with a dense selective layer and a porous sub-layer with finger-like structures. The DES-modified membranes (M_2_-M_10_) showed smoother and more regular surface morphology than the pristine PEI membrane. However, increasing modification time in the M_7_-M_10_ membranes provided lower surface smoothness due to DES-aggregation on the membrane surface, which could result in obstruction of the membrane surface pores. It should be mentioned that M_6_'s regular porous sub-layer was observed compared to the others, which indicates that the PEI matrix was at least affected by surface modification. Furthermore, the SEM images of the bare (M_1_) and optimally modified (M_6_) membranes were presented in Fig. 4 to highlight the effect of DES on the membrane morphology.

Supplementary 2 depicts 3D AFM scans from the top perspective of DES-PEI membranes. The light and dark areas in the AFM images demonstrate peaks and valleys on the membranes' surface, respectively. Table 3 tabulates the roughness parameters of the membranes' surface, i.e., mean roughness (S_a_), root average square of the Z data (S_q_), and mean difference of the highest and lowest regions (S_z_). In overall, the treated membranes had a smoother surface due to the hydrogen bond development between DES and PEI and also the DES hydrophilic nature. As can be seen, the surface roughness of the pristine PEI membrane was reduced from S_a_ = 19.23 nm to S_a_ = 10.25 nm due to coating with DES composed of Ch/Gu ratio of 0.5 at 30 h (M_6_). However, increasing the modification time and Ch/Gu ratio led to a rise in the peak intensity and valley depth, so S_a_ showed an ascending trend for M7-M10. The highest S_a_ was reported as 15.24 nm for M_10_ with the highest modification time and Ch/Gu ratio (48 h and 2). This incremental tendency in surface roughness might be owing to DES and PEI incompatibility caused by increased agglomeration at further modification times and Ch/Gu ratios. Since foulants are highly inclined to adhere to a rougher surface, there is a clear link between antifouling properties and membrane surface roughness [28]. Accordingly, the used DES can be considered as a surface-modifying agent for PEI membranes for improving the smoothness and morphology of the surface.

**Table 3:** The surface roughness parameters of the prepared membranes.

**Membrane**	**S**_**a**_**(nm)**	**S**_**q**_**(nm)**	**S**_**y**_**(nm)**
M_1_	19.23	20.19	24.32
M_2_	16.80	21.19	113.25
M_3_	12.92	12.90	82.94
M_4_	13.55	16.80	115.13
M_5_	14.95	19.19	119.76
M_6_	10.25	14.26	150.20
M_7_	13.79	16.49	90.07
M_8_	18.61	22.94	159.59
M_9_	12.24	14.80	82.80
M_10_	15.24	19.47	139.15

### 3.2 Modeling of filtration process

The simultaneous impacts of two independent variables (Ch/Gu ratio (A) and modification time (B)) were evaluated on the filtration performance by applying RSM through a central composite design (CCD) regression model. Optimization of the membrane modification condition was conducted on membrane performance in terms of four responses: PWF (L/m^2^.h), FRR (%), R_ir_ (%), R_t_ (%); the results are represented in Table 4. Accordingly, the results of the analysis of variance (ANOVA) and the coded models of the evaluated responses are tabulated in Table 4.

**Table 4:** ANOVA results obtained for the studied responses.

**Response**	**Modified equations with significant terms**	**Probability**	**R**^**2**^	**Adj. R**^**2**^	**S.D**	**CV**	**lack of fit**
PWF	11.2357–2.43667A + 0.591667 B −1.2675 AB -3.07738 B^2^	0.0003	0.9121	0.8682	0.9487	9.67	0.1860
FRR	91.5271–2.515A -1.56667 B −8.33048 B^2^	<0.0001	0.9242	0.8989	1.59	1.81	0.7957
R_ir_	7.78 + 2.515 A + 1.565 B + 9.015 B^2^	<0.0001	0.9830	0.9774	0.7778	6.51	0.0409
R_t_	2.35429 × 10^+13^ + 6.15 × 10^+12^ A −2.08333 × 10^+12^ B +1.12071 × 10^+13^B^2^	<0.0001	0.8942	0.8589	2.94 × 10^12^	10.25	0.0006

#### 3.2.1 Pure water flux (PWF)

ANOVA data were calculated for the PWF response to investigate the influence of variables on membrane performance and the designation of the suggested model consistency. A quadratic model as a function of the studied variables on PWF with significant terms was specified as shown in Table 4. According to the provided model, the determination coefficient values, including R^2^ and adjusted R^2^, were acquired to be 0.9121 and 0.8682, respectively, indicating the good fitness of the regression model.

**Fig. 5:**
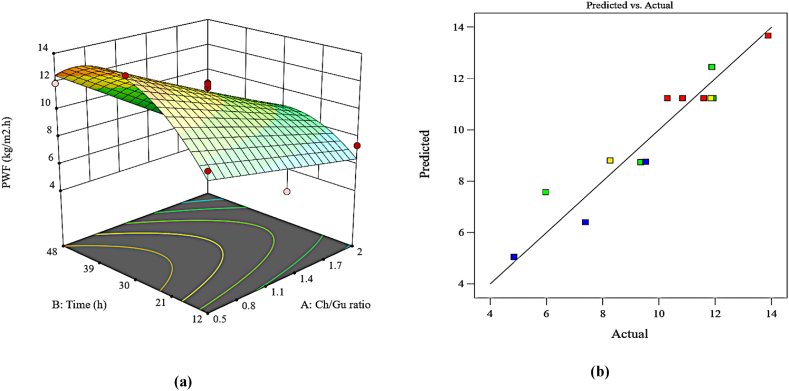
Plots of (a) 3D surface (b) predicted vs. actual values for PWF response.

**Fig. 6:**
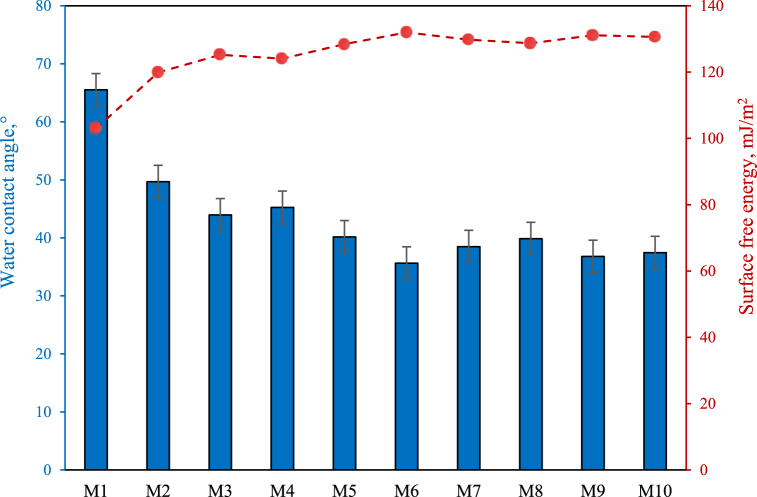
Water contact angles (WCA) and surface energies (-ΔGs) of the DES-PEI membranes.

Fig. 5a demonstrates the 3D surface of the synchronic effects of the Ch/Gu ratio and modification time of the membrane on the PWF response. According to Fig. 5a, the variables represented different impacts. Increasing the modification time to 30 h had a positive impact on PWF due to effective surface modification. This was confirmed by the water contact angle (WCA) results (Fig. 6). The modification of the PEI membrane surface using the hydrophilic DES resulted in a decrease in the contact angle of the PEI membranes from 65.5° (pristine PEI) to 35.63° (M_6_), indicating an enhancement in the hydrophilicity of the PEI surface. The surface energy values (-ΔGs), representing how hydrophilic a membrane's surface is, further confirmed the improved hydrophilicity (Fig. 6). When the PEI membrane surface is modified with the hydrophilic DES, the WCA of the modified membranes decreases, and the -ΔGs values show an increasing trend, indicating enhanced hydrophilicity of the PEI membrane surface. Despite the WCA decreasing up to 30 h, the WCA increased at 48 h of modification time due to DES aggregation and incompatibility at more than optimal times. It is obvious that this trend was also observed in the PWF response. Within a span of 48 h, the detrimental effect of the change in time on PWF was mostly caused by an elevated level of DES-aggregation on the surface of the membrane, resulting in the blockage of the membrane's pores. Also, it's worth mentioning that the lowest Ch/Gu ratio with 30 h modification time (M_6_) was found to present the highest PWF value (13.88 L/m^2^.h) and the lowest WCA values, where the membrane had the most negative surface charge (zeta potential results presented in Fig. 3). Besides, the actual plot vs. predicted PWF response is depicted in Fig. 5b, showing perfect agreement between experimental data and predicted ones.

#### 3.2.2 Flux recovery ratio (FRR)

**Fig. 7:**
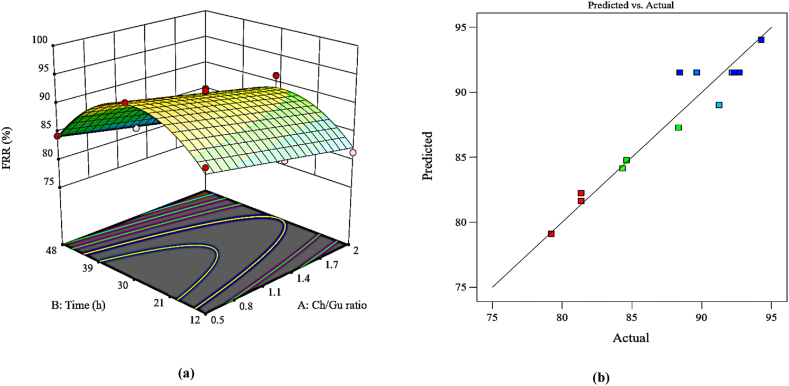
Plots of (a) 3D surface (b) predicted vs. actual values for FRR response.

A quadratic polynomial model with significant coded terms and a confidence level of more than 95 % (p-value <0.0001) was provided for the FRR response, defined at Table 4. The model validation was corroborated by calculating the values of R^2^ (0.9242), and adjusted R^2^ (0.8989). Fig. 7 indicates the 3D surface plot of the variables' concurrent influence on the FRR response. Similar to PWF, increasing the modification time of the membrane up to 30 h resulted in increasing FRR caused by ameliorated surface modification and, subsequently, hydrophilicity enhancement (WCA and -ΔGs result) and improved surface morphology (AFM result). Nevertheless, the reduction of FRR over subsequent modification times was attributed to pore blocking and the development of a cake layer on or inside the membrane due to its poor hydrophilicity and rough surface. The high FRR was achieved with a low Ch/GU ratio, which is plausible because GU has a more negative surface and higher hydrophilicity compared to Ch, The maximum value of FRR (94.25 %) was achieved at 30 h with a Ch/Gu ratio of 0.5 (M_6_), while the minimum value (79.21 %) was obtained at 48 h with a Ch/Gu ratio of 2 (M_10_), indicating the influence of studied variables on FRR response. The accordance between the actual values measured and the predicted values for FRR is also shown in Fig. 7b.

#### 3.2.3 Irreversible fouling (R_ir_)

The R_ir_ response was measured to investigate the antifouling capacity of the DES-PEI membranes. The p-value of <0.0001, R^2^ of 0.9830, and the adjusted R^2^ of 0.9774, signify the perfect regression model. The suggested quadratic model for this response, which is a function of two effective variables on membrane modification, was specified as represented in Table 4.

**Fig. 8:**
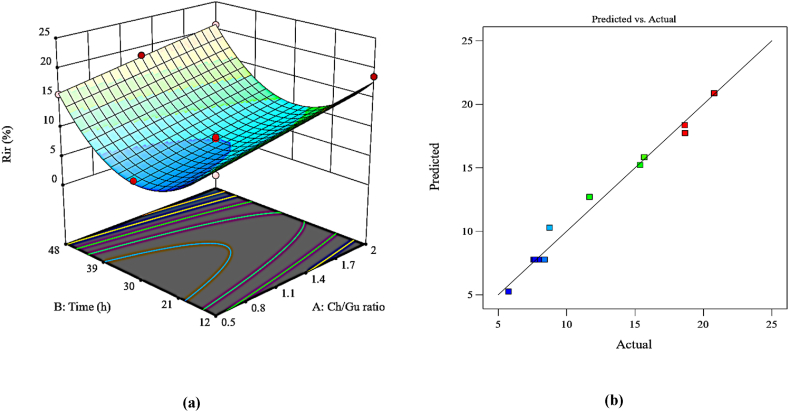
Plots of (a) 3D surface (b) predicted vs. actual values for R_ir_ response.

The 3D surface plot in Fig. 8a shows the concurrent impacts of variables on the R_ir_ response. The results indicated a more obvious effect of the membrane modification time compared to the Ch/Gu ratio. The low R_ir_ value was obtained with a low Ch/Gu ratio and a moderate modification time ranging from 5.75 % to 20.78 %. These findings were in line with the WCA, Zeta potential, and AFM investigations mentioned previously. Organic foulants penetrate into the surface valleys of the membrane, making rougher surface more susceptible to foulant adsorption and elevating Rir. On the other hand, hydrophilic DES induces an enhancement in membrane surface wettability, mitigating foulant accumulation via the development of a dense and stable hydration layer. However, as previously noted, the reduced antifouling capability at high Ch/Gu ratios and extended modification times was attributed to the positive characteristics of Ch in comparison to Gu and the additional agglomeration of DES. There was a good correlation between the actual and predicted values of the Rir response shown in Fig. 8b.

#### 3.2.4 Total resistance (R_t_)

**Fig. 9:**
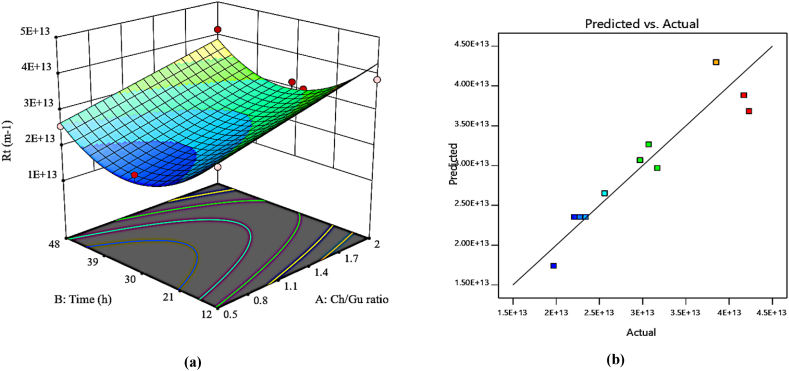
Plots of (a) 3D surface (b) predicted vs. actual values for R_t_ response.

A quadratic model was proposed to analyze the effects of the two evaluated variables. A p-value of <0.0001 (a confidence level of more than 95 %) signifies an appropriate regression model (Table 4). According to the suggested equation, A, B, and B^2^ were effective model terms on R_t_, indicating further influence of modification time on the response of the membranes. Fig. 9a shows the simultaneous effects of the Ch/Gu ratio and modification time variables on the response. R_t_ value in all DES-PEI membranes experienced a diminishment compared to the pristine membrane. This decline was observed up to 30 h of the modification time at a 0.5 Ch/Gu ratio. The intrinsic resistance of PEI membranes allows them to have good fouling resistance in general, however, enhanced antifouling properties were observed in the PEI membrane decorated by DES, thanks to its smoother surface and increased hydrophilicity. According to the aforementioned reasons, the lowest membrane resistance was obtained at a 30-h modification time and a minimum Ch/Gu ratio value. Fig. 9b illustrates the actual plot vs. predicted R_t_ response, revealing a befitting agreement between the experimental data and the predicted ones.

### 3.3 Optimization of the process and verification

**Fig. 10:**
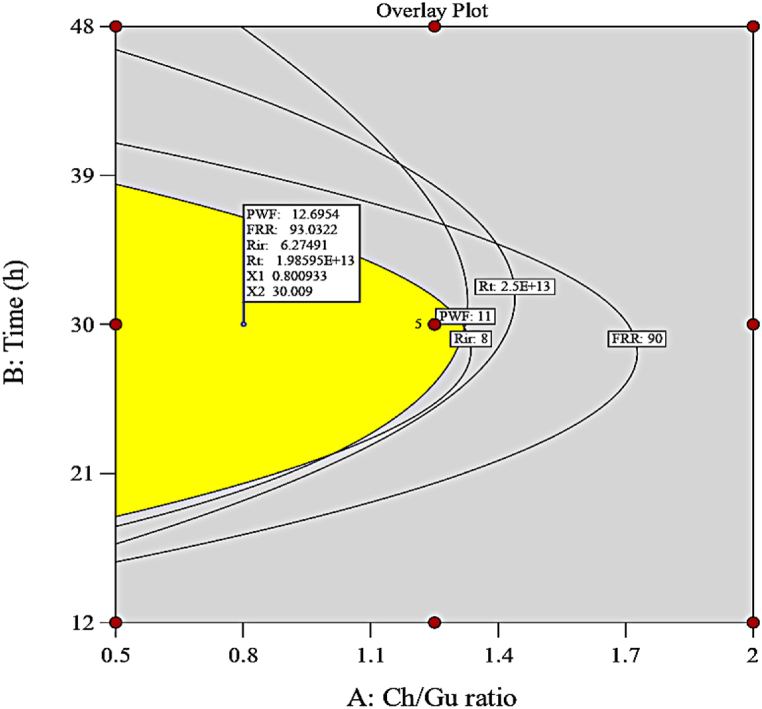
Overlay plot for the optimization of the filtration process.

The goal of the optimization process is to achieve an experiment condition that encompasses the optimal ranges of variables, resulting in the membrane filtration performance reaches its maximum value. A graphical optimization was employed to predict the optimal performance of the DES-PEI membrane within the design ranges of the studied variables. Fig. 10 shows the graphical optimization for the performance of the DES-treated PEI membrane, in which the shaded portion indicates the values of the conceivable four evaluated responses (PWF, FRR, R_ir_, R_t_) in the variable space. The results highlighted that the suggested model predicted the best performance of the DES-PEI membrane under optimum conditions, including a Ch/Gu ratio of 0.8 and a modification time of 30 h. Also, a membrane was fabricated with Ch/Gu ratio of 0.8 and a modification time of 30 h to validate the predicted optimal condition obtained from the models and its PWF, FRR, and Rir were measured. Both the predicted and experimental values of each response were reported in Table 5. According to the Table, standard deviation was in the range of 0.27–1.92 indicates a good agreement between the predicted and experimental values, corroborating that the used models were able to accurately predict performance.

**Table 5:** Verification experiments under the optimum condition.

**Membranes**	**Conditions**		**Response**		
			**PWF, L/m**^**2**^**.h**	**FRR,%**	**Rir, %**
M_11_	Ch/Gu ratio = 0.8 Time = 30 h	Experimental values	11.51	95.74	5.89
		Model values	12.69	93.03	6.27
		Standard deviation	0.83	1.92	0.27

### 3.4 Rejection performance evaluation

**Fig. 11:**
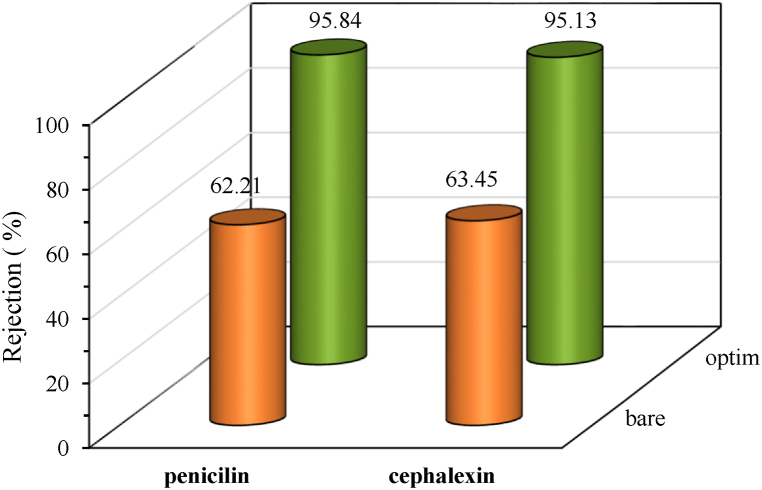
Performance comparison of the bare (M_1_) and the fabricated optimal DES/PEI (M_11_) membranes for penicillin and cephalexin antibiotics rejection.

Antibiotics are the main environmental concern in wastewater treatment technology and filtration process is a successful technique to remove them from wastewater among other treating techniques. Therefore, the effectiveness of DES-coating on PEI membrane performance was evaluated using two antibiotics, penicillin and cephalexin, in the present research. The rejection performance of the bare (M_1_) and the fabricated membrane based on the obtained optimal condition (M_11_) was evaluated by using penicillin and cephalexin antibiotics, which the obtained results presented in Fig. 11. The penicillin and cephalexin rejections of the bare membrane were found to be 62.21 and 63.45 %, respectively. Although, the rejection of the optimally modified membrane presented a significant enhancement so that it reaches 95.84 and 95.13 % for penicillin and cephalexin, respectively. For both the antibiotics, the optimal membrane had a 34 % increase in rejection overall.

To come up with an acceptable explanation for this outcome, it's worth noting that the combination functions of steric and Donnan effects contribute to the variations in the membrane's rejections [29]. Since the pore size of the PEI membrane surface decreased by coating Ch/Gu DES solution, Donnan effects may play a dominant role in antibiotic separation. Both the penicillin and cephalexin antibiotics have some common OH, C=O, and N-H groups, which could be rejected by hydrophilic functional groups of DES and more negative charge on the surface (see Fig. 2). Also, the molecular weight may play a role in why there is no significant difference in the rejection of two antibiotics in optimally modified membranes which the molecular weight of penicillin and cephalexin being very close to each other (350.39 and 347.39 g/mol, respectively) [30].

### 3.5 Long-term performance assessment

**Fig. 12:**
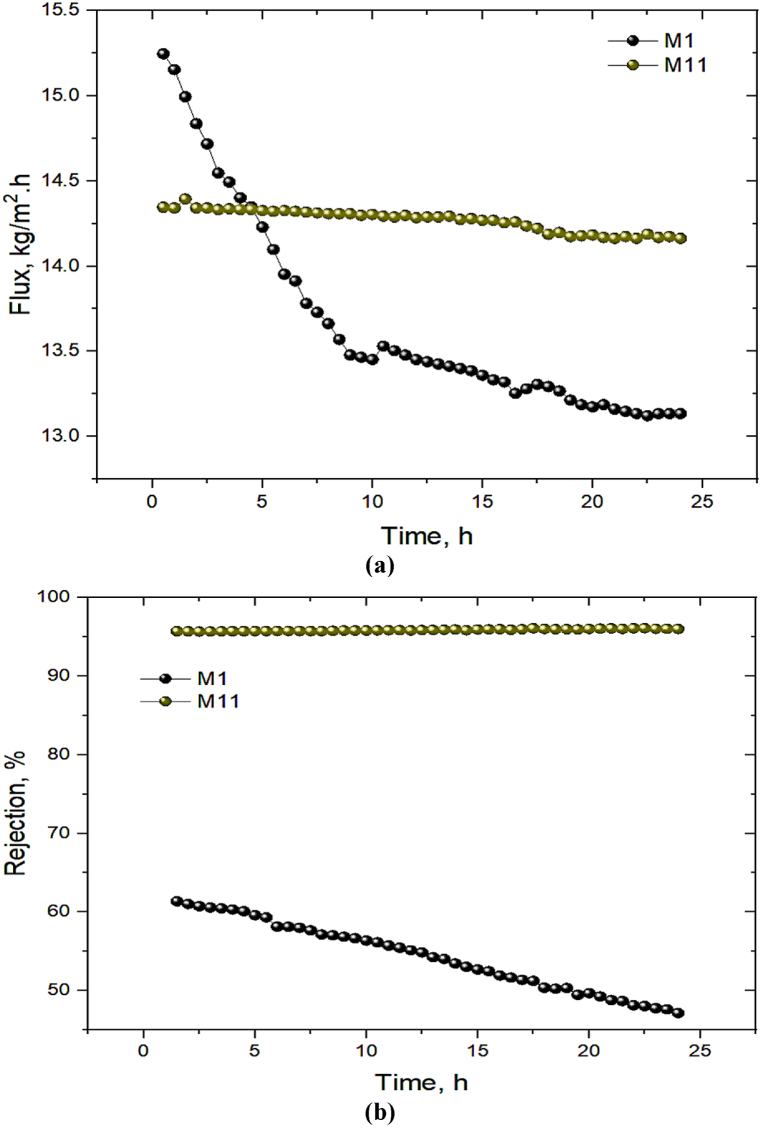
Long-term filtration performance in terms of (a) flux and (b) rejection of penicillin antibiotic for the bare (M_1_) and DES/PEI (M_11_) membranes in the dead-end setup.

Due to the similar rejection data of penicillin and cephalexin, penicillin was selected to assess long-term performance of the fabricated membranes. The 0.01 gr/l penicillin antibiotic solution was fed for 24-h long-term filtration in a dead-end setup. In order to evaluate and compare filtration capacity, the flux and rejection efficiency of the bare (M_1_) and optimally modified membranes (M_11_) were measured and presented in Fig. 12. At the beginning of filtration, the flux of M_11_ (14.34 L/m^2^.h) was lower than that of M1 (15.24 L/m^2^.h) as a result of the nature of surface modification technique, which reduces PWF. However, the flux profile during a 24-h filtration for the bare PEI membrane shows a flux reduction from 15.24 to 13.13 L/m^2^.h. In contrast, a stability in filtration behavior was observed for M_11_ from 14.34 to 14.16 L/m^2^.h. The rejection data showed similar results. As shown in Fig. 12b, antibiotic rejection for M1 was decreased by about 15 % during a 24-h filtration. However, M_11_ presented a stable rejection of 96 %, confirming the improved hydrophilic and antifouling properties. The conclusion is that DES is an effective coating layer, as it was found that the PEI membrane coated with DES had an increased resistance to fouling and increased flux stability.

**Fig. 13:**
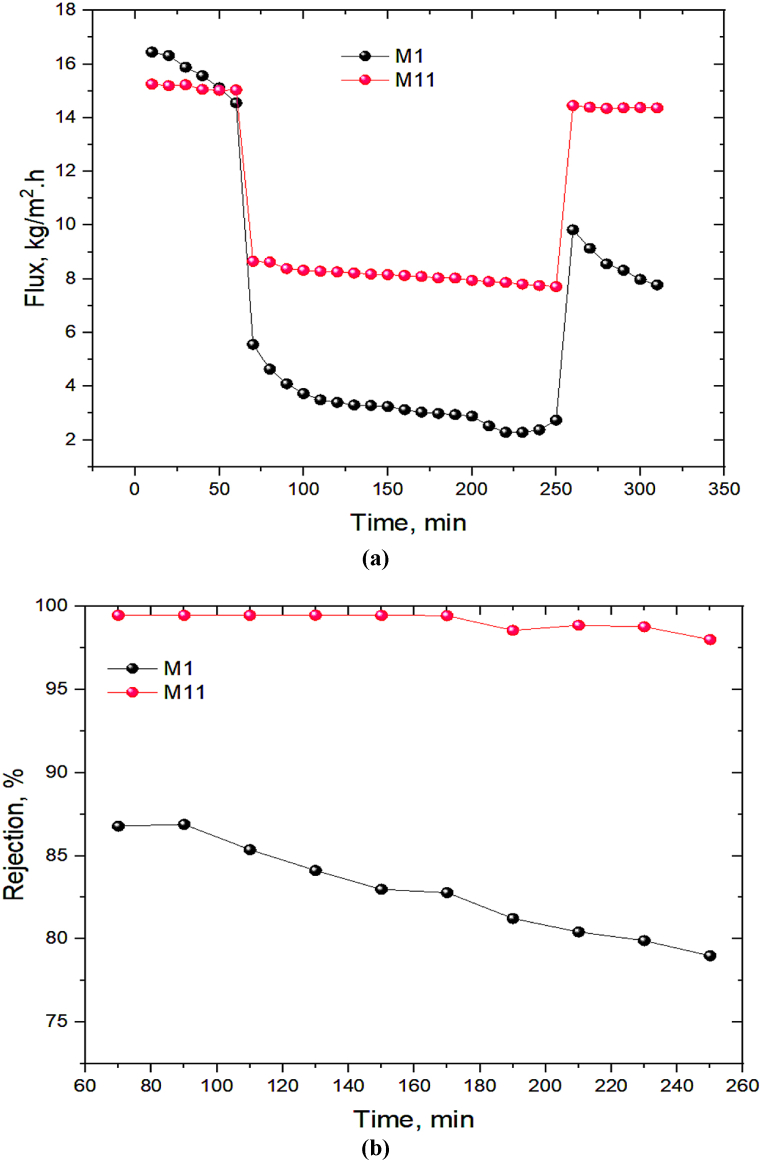
Long-term filtration performance in terms of (a) flux and (b) rejection of penicillin antibiotic for the bare (M_1_) and DES/PEI (M_6_) membranes in the cross-flow setup.

The comparison of the long-term filtration process for the bare (M_1_) and DES/PEI optimum membranes (M_11)_ was also conducted in the cross-flow system utilizing penicillin antibiotic at a flow rate of 120 L/h and pressure of 4 bar. It should be mentioning that the cross-flow filtration was performed in three step filtration including the first pure water filtration, penicillin filtration, and the second pure water filtration. From Fig. 13a, M_1_'s PWF at the beginning of filtration was higher than M_11_, similar to the dead-end set up. Over the penicillin filtration step, M_11_'s flux showed a slight decrease from 8.6 to 7.71 L/m^2^.h, whereas M_1_'s flux has gradually decreased from 5.56 to 2.38 L/m^2^.h.

Besides, according to Fig. 13b, a long-term filtration displayed that M_11_'s rejection remained ideally high and stable (from 99.47 to 98.56 %), whereas M1 had a 11.5 % drop in rejection (from 86.78 to 75.25 %). Furthermore, M_11_ is much more capable of recovering flux after fouling compared to M1 as observed in Fig. 13a. The DES coating on the PEI membrane surface is responsible for the improvement and stability of rejection behavior and permeation of M11. Theoretically, super hydrophilic DES develops a hydration layer on the membrane surface by hydrogen bonding water molecules, it boosts the flux and strengthens membrane resistance to cake layer formation, resulting in increased flux and rejection in long-term performance.

### 3.6 Comparative study of the literature

To compare the results obtained from the current study with existing literature, information from several studies that utilized DES as a modifying agent has been summarized and presented in Table 6. From the reported results, various polymeric membranes have been modified by DES using different precursors, as shown in Table 6. Overall, all the polymeric membranes demonstrated high performance in terms of PWF, rejection, and FRR. PWF from other studies is higher than that of the current research using PEI, which is related to the characteristics of polymeric PEI, resulting in low PWF. On the other hand, all the membranes demonstrated high performance in rejecting various solutes, primarily exceeding 90 %, which indicates the significant potential of the membranes in the rejection process. Furthermore, by comparing the FRR data presented in Table 6, it can be concluded that the DES utilized in this study demonstrated a superior potential for recovering permeate flux, achieving 95.74 % FRR. In conclusion, DES containing Choline Chloride/Guanidine exhibits remarkable hydrophilicity, fouling resistance, and rejection potential in the polymeric membrane due to its hydrophilic properties.

**Table 6:** Comparison of the performance of the mixed matrix DES/PVDF membrane with reported DES blended polymeric membranes.

**Membrane type**	DES composition	PWF (L/m^2^.h (operating pressure)	Selectivity (rejection,%)	FRR (%)	Ref.
PES (15 wt%)	Decanoic acid: N4444-Cl (2:1)	71.4 (2 bar)	BSA: 99.0	76.4	[31]
PES (20 wt%)	Choline chloride: Lactic Acid (1:9) and lignin	81.5 (3 bar)	PEG 35 kDA: 85.7	–	[32]
PVDF, PAN (10 wt%)	Phenyl acetic acid: trimethyl glycine (2:1)	818.89 (3 bar)	Methylene blue:54	–	[33]
PSF (17 wt%)	Choline Chloride: D-Fructose	12 (1 bar)	Stormwater: 99	54	[34]
PES (18 wt%)	Choline Chloride: Ethyl Glycol (1:2)	80.4 (3 bar)	BSA: 98.9 Dye: 99.2	70.6	[23]
PSF	Choline Chloride-urea (1:2)	56.7	NaCl: 96.4	–	[9]
PEI	Choline Chloride/Guanidine (0.8:1)	11.51 (3 bar)	Penicillin: 95.84	95.74	This work

## 4 Conclusion

In summary, efficient decoration of the PEI membrane surface was conducted using DES, a mixture of choline chloride (Ch) and guanidine (Gu), by considering two functional variables including the Ch/Gu ratio and modification time. From the obtained results, the surface morphology, roughness, surface charge, wettability, as well as surface free energies of the DES-decorated PEI membranes, were all affected by coating. The performance of the treated membranes was investigated by measuring four responses, i.e., PWF, FRR, R_ir_, and R_t_. The values of PWF, FRR, R_ir_, and R_t_ were found to be 12.67 L/m^2^.h, 92.99 %, 6.31 %, and 1.99 e+13 %, respectively, at the optimum modification condition comprising a 0.8 Ch/Gu ratio and a 30-h modification time. In the next step, the rejection of penicillin in both the dead-end and cross-flow set ups over long-term filtration was monitored. The results showed a significant and stable rejection of the optimally modified membrane (higher than 96 %), however, the bare membrane showed a decreasing trend in rejection over filtration (between 45 and 60 %). Overall, this study, in addition to optimizing of modification conditions, introduced DES as a multitasking agent to modify the surface roughness and hydrophilicity of PEI membrane to boost PWF and also improved antifouling and rejection properties in long-term filtration.

## CRediT authorship contribution statement

**Foad Gholami:** Data curation, Conceptualization. **Azar Asadi:** Writing – review & editing, Methodology, Funding acquisition. **Mina Dolatshah:** Writing – original draft, Data curation. **Safoora Nazari:** Writing – review & editing, Software.

## Declaration of competing interest

The authors declare that they have no known competing financial interests or personal relationships that could have appeared to influence the work reported in this paper.

## Footnotes

Appendix A Supplementary data to this article can be found online at https://doi.org/10.1016/j.heliyon.2024.e41432.
