# Supplementary material for: Modeling and optimization of surface modification process of ultrafiltration membranes by guanidine-based deep eutectic solvent
Source: Heliyon. 2024 Dec 21;11(1):e41432. doi: 10.1016/j.heliyon.2024.e41432 (PMC11732549; doi:10.1016/j.heliyon.2024.e41432)
Supplement: Multimedia component 1 [file mmc1.docx]

|  | Surface | Cross |
| --- | --- | --- |
| M_1_ | 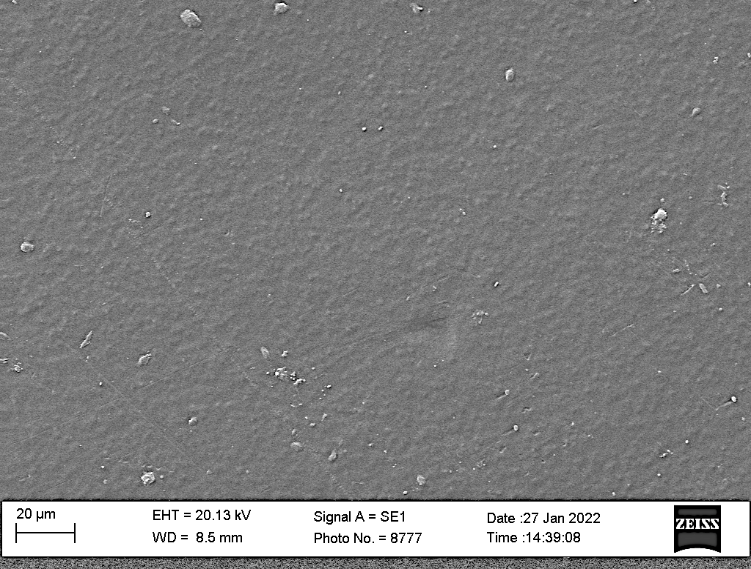 | 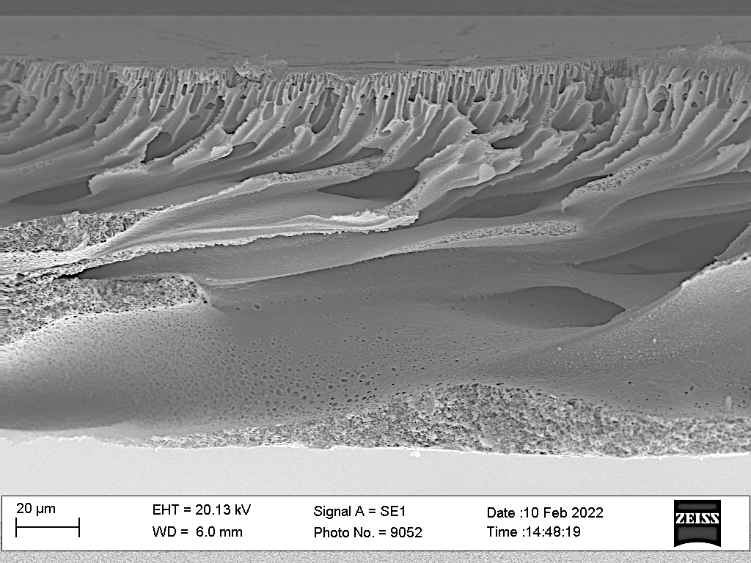 |
| M_2_ | 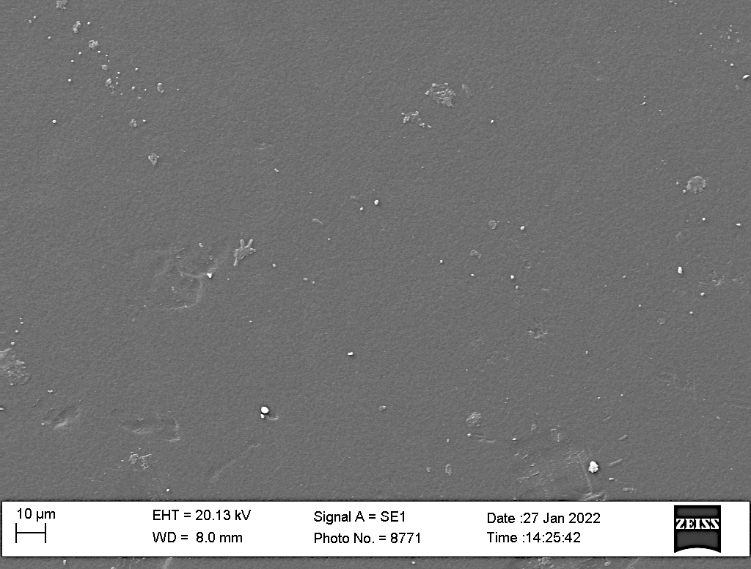 | 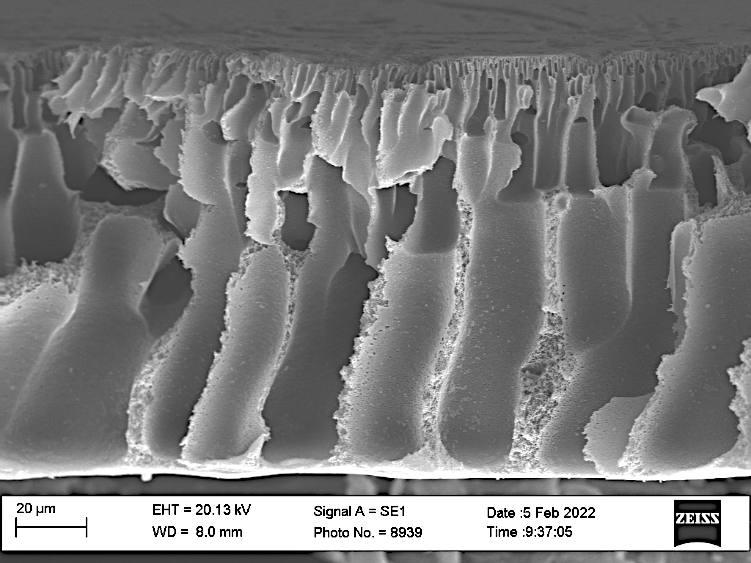 |
| M_3_ | 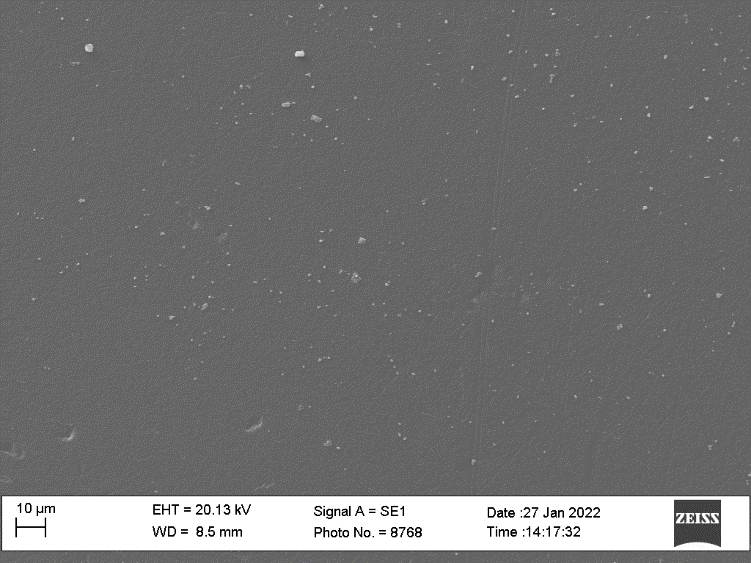 | 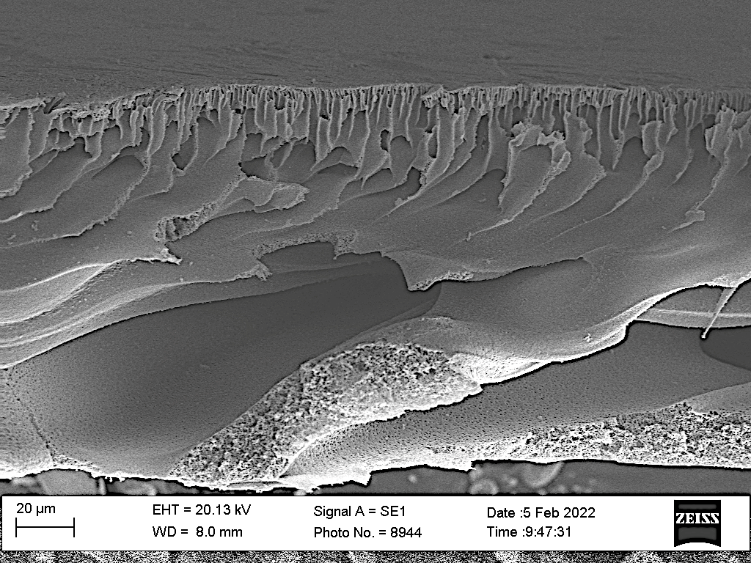 |
| M_4_ | 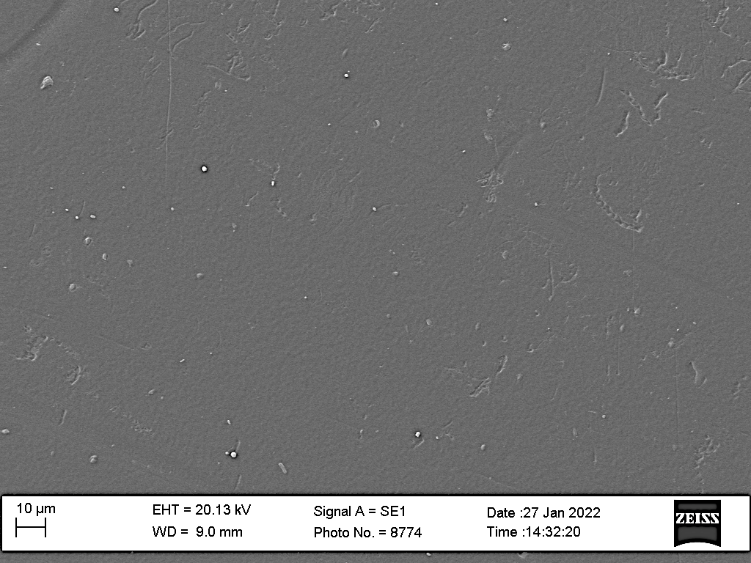 | 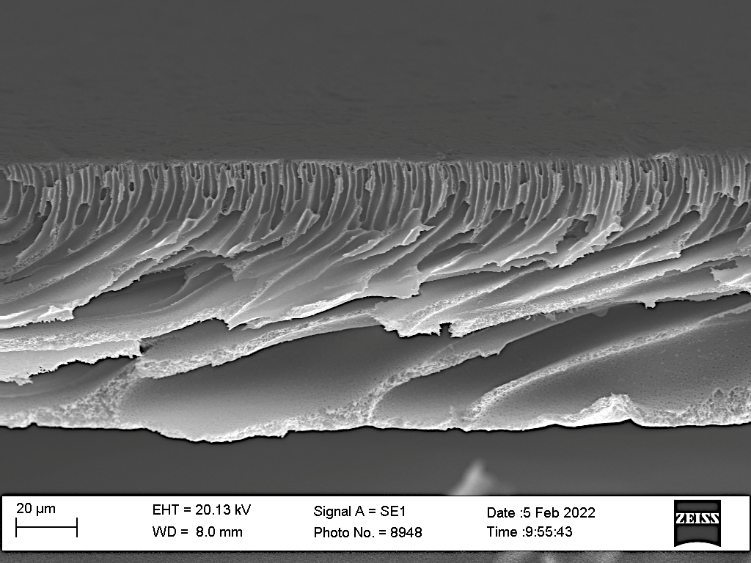 |
| M_5_ | 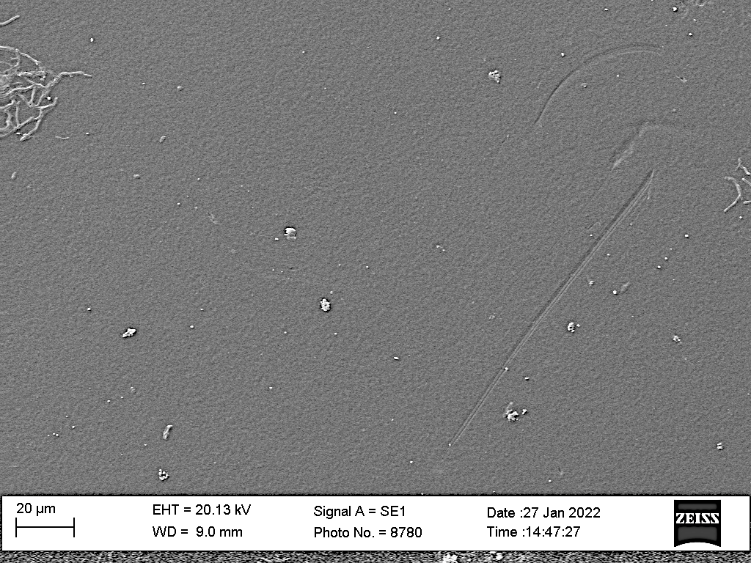 | 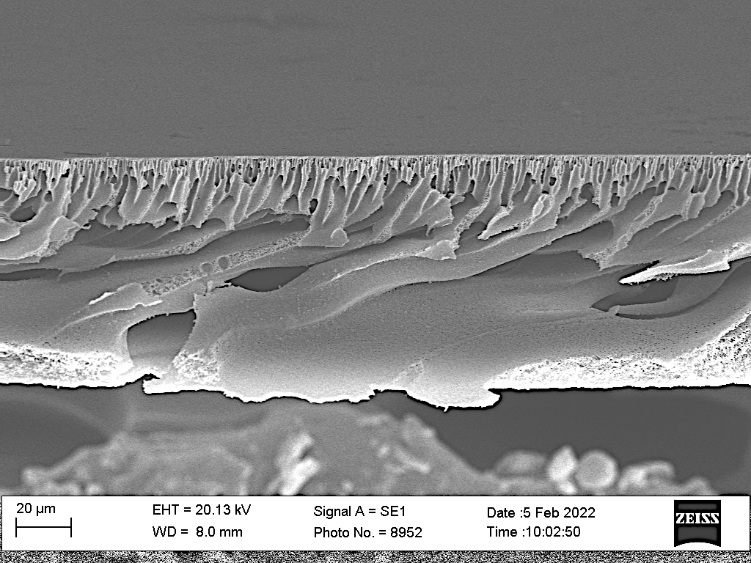 |
| M_6_ | 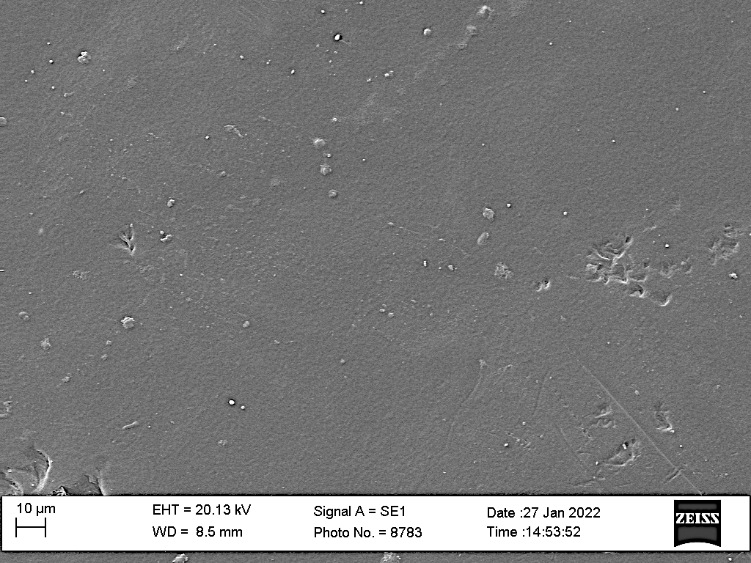 | 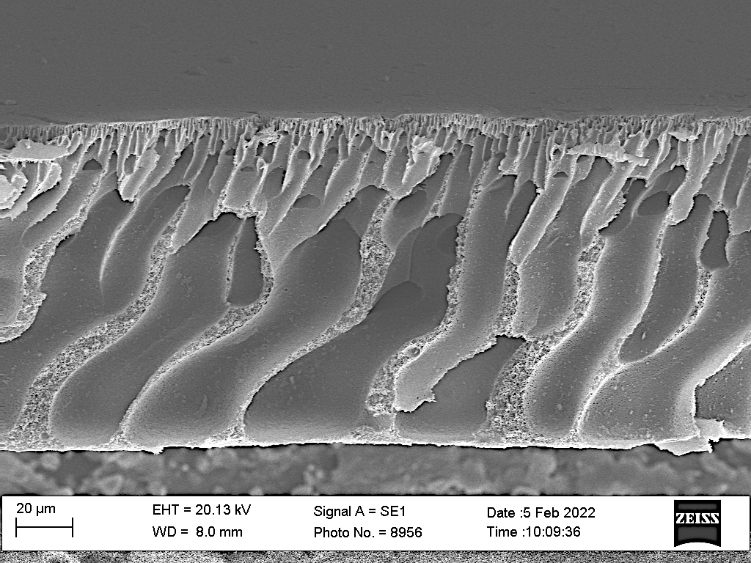 |
| M_7_ | 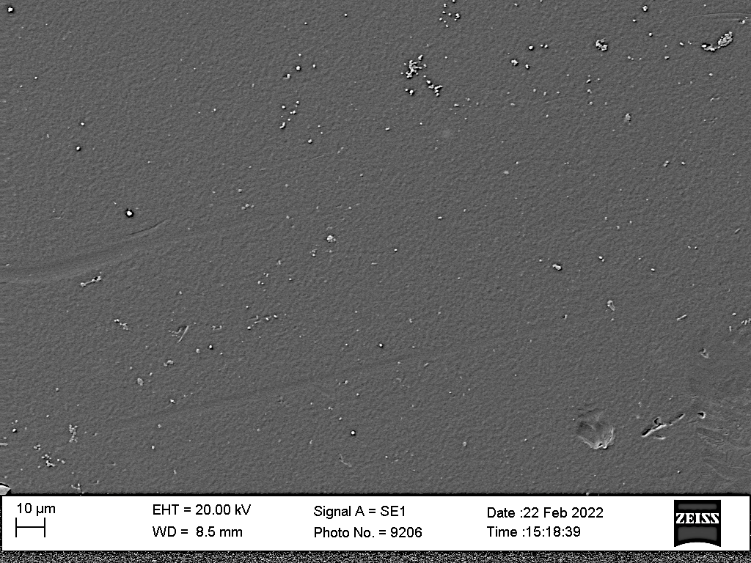 | 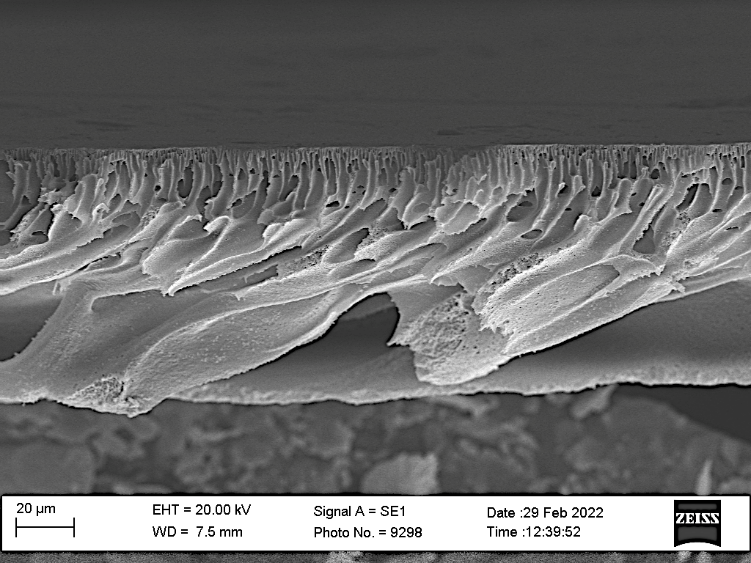 |
| M_8_ | 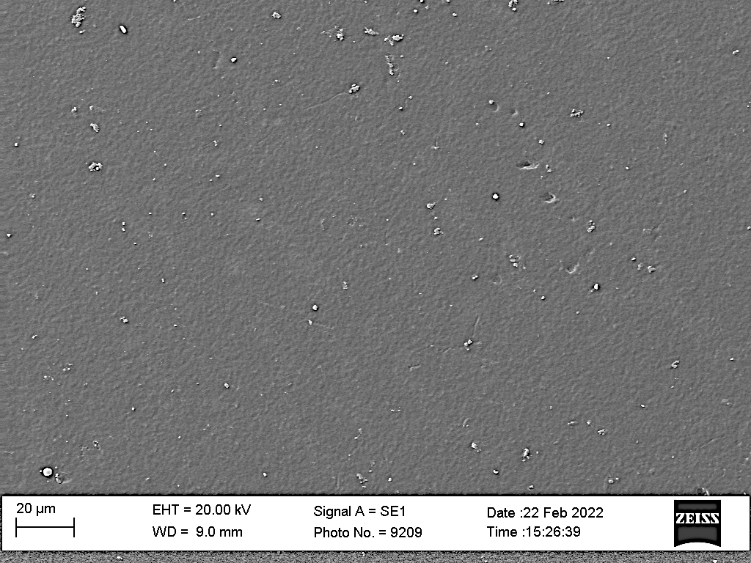 | 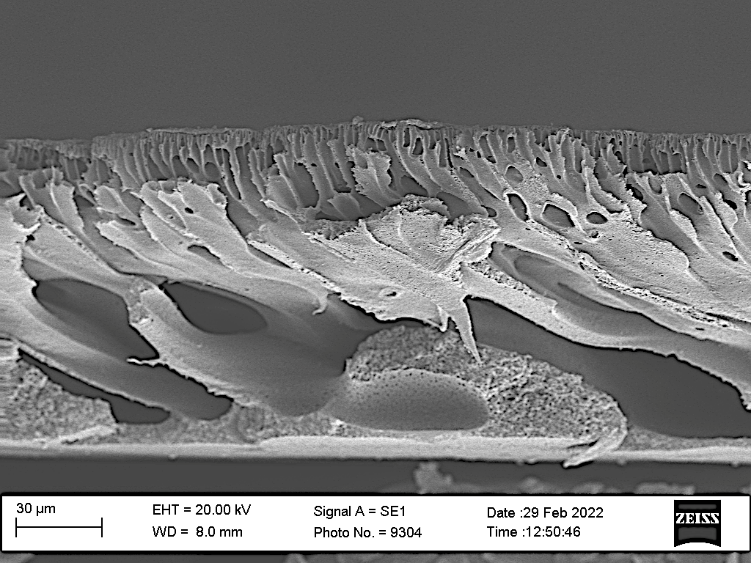 |
| M9 | 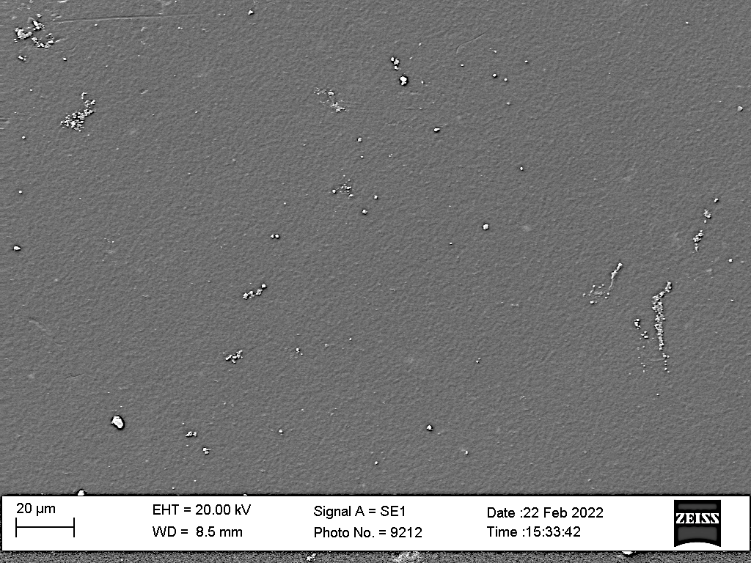 | 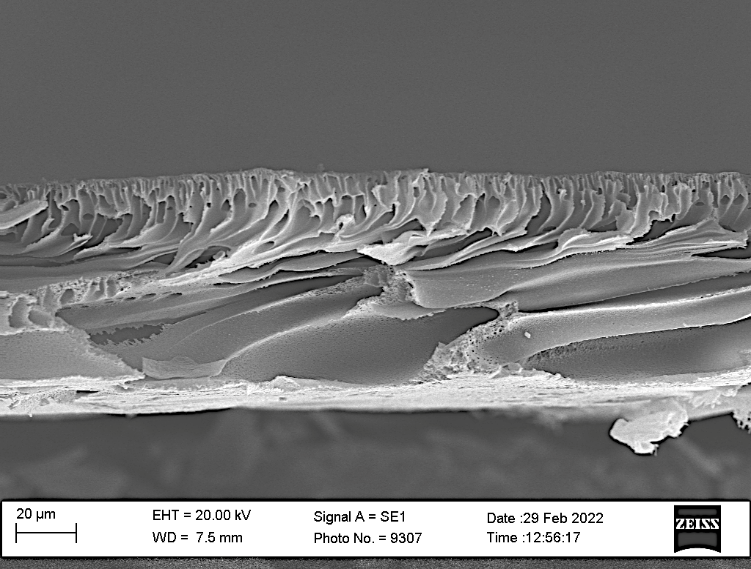 |
| M_10_ | 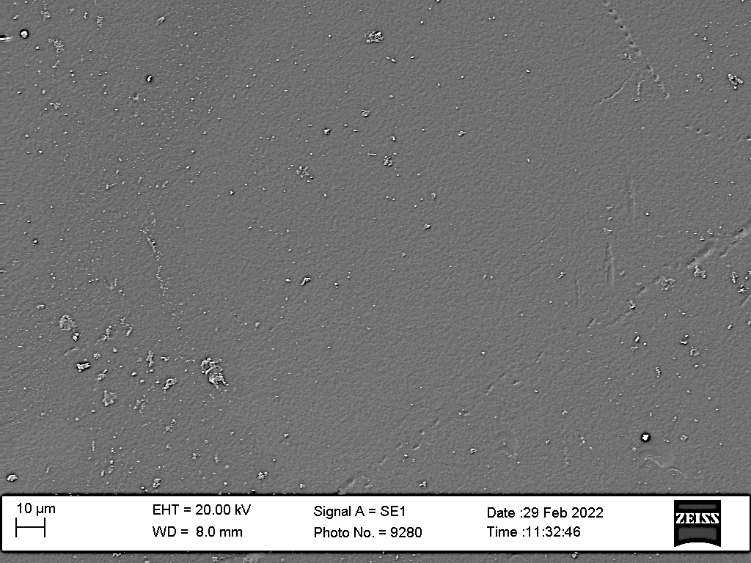 | 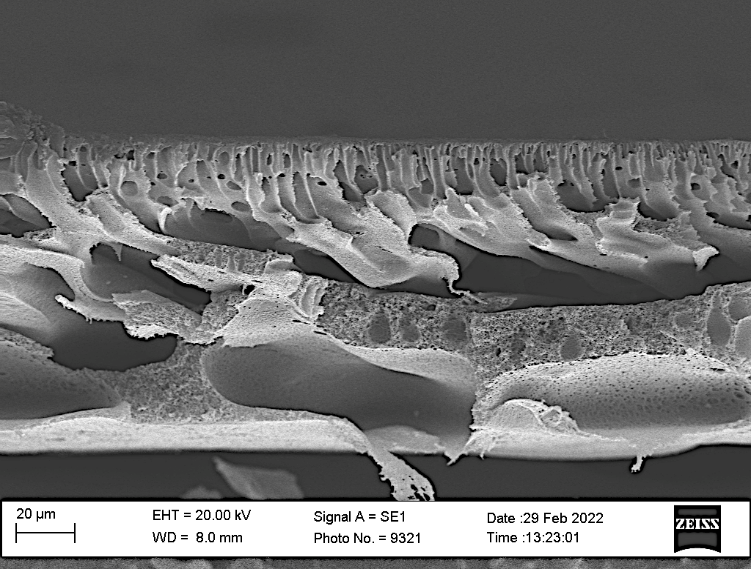 |
| Supplementary Fig. 1. Surface and cross-section SEM images of the DES-PEI membranes. | | |

| 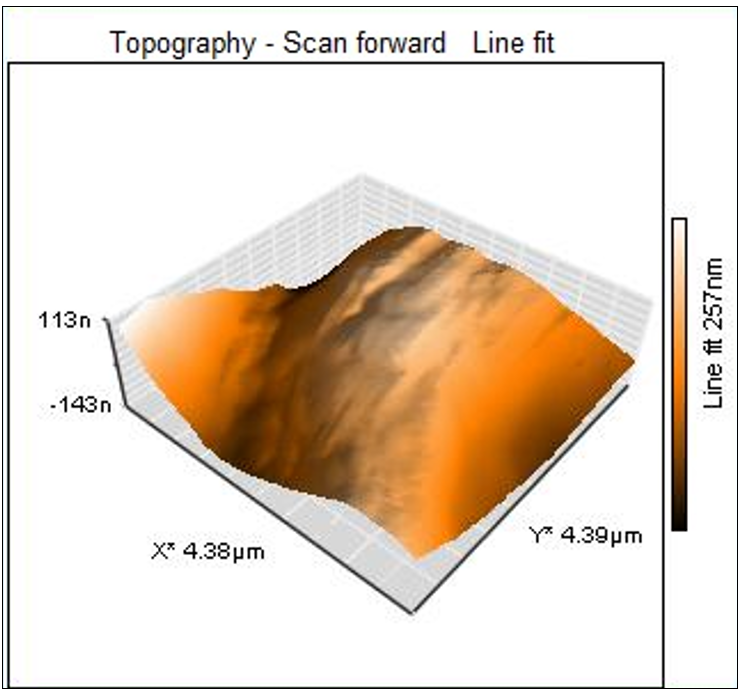 | 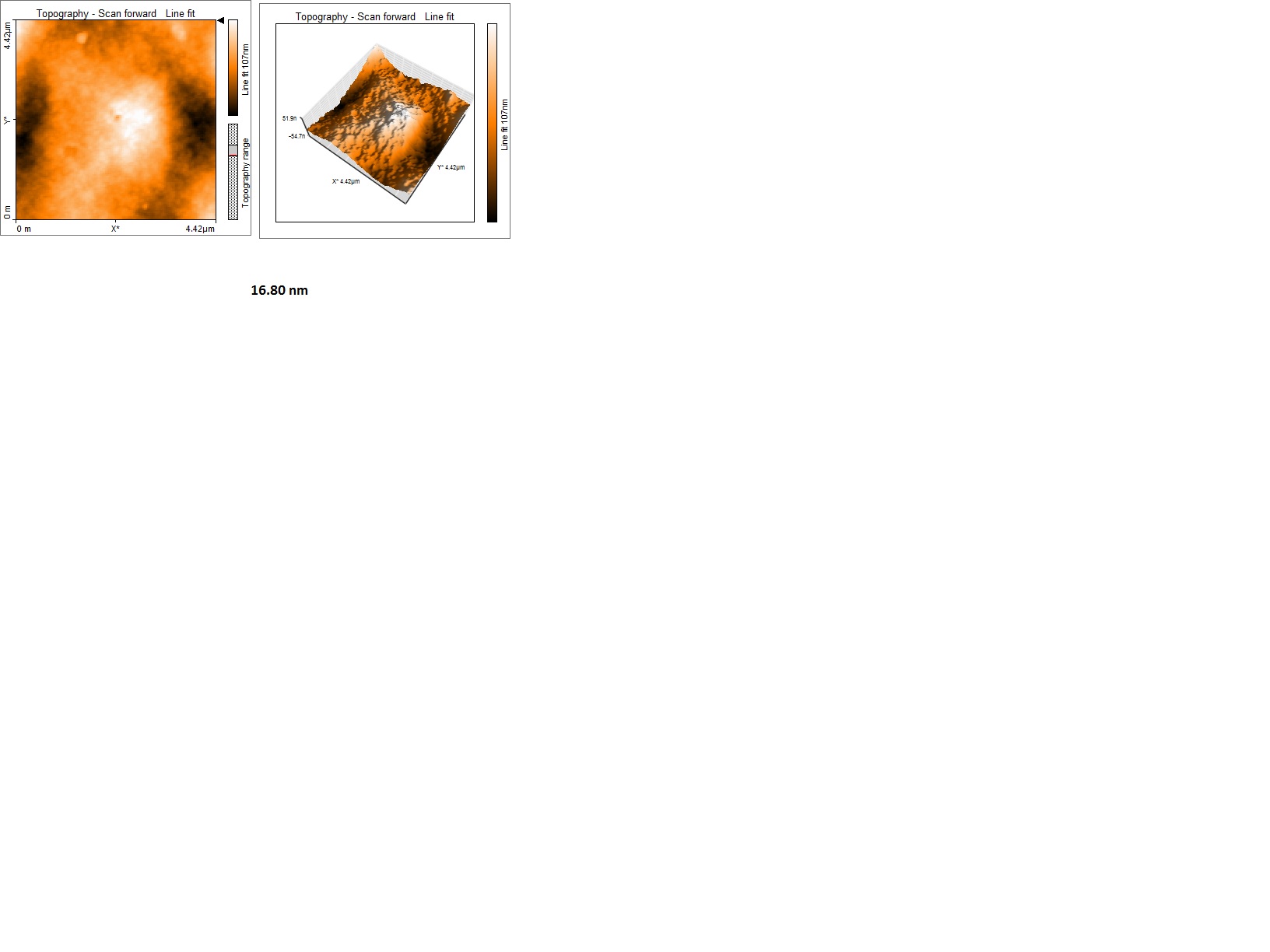 |
| --- | --- |
| M_1_ | M_2_ |
| 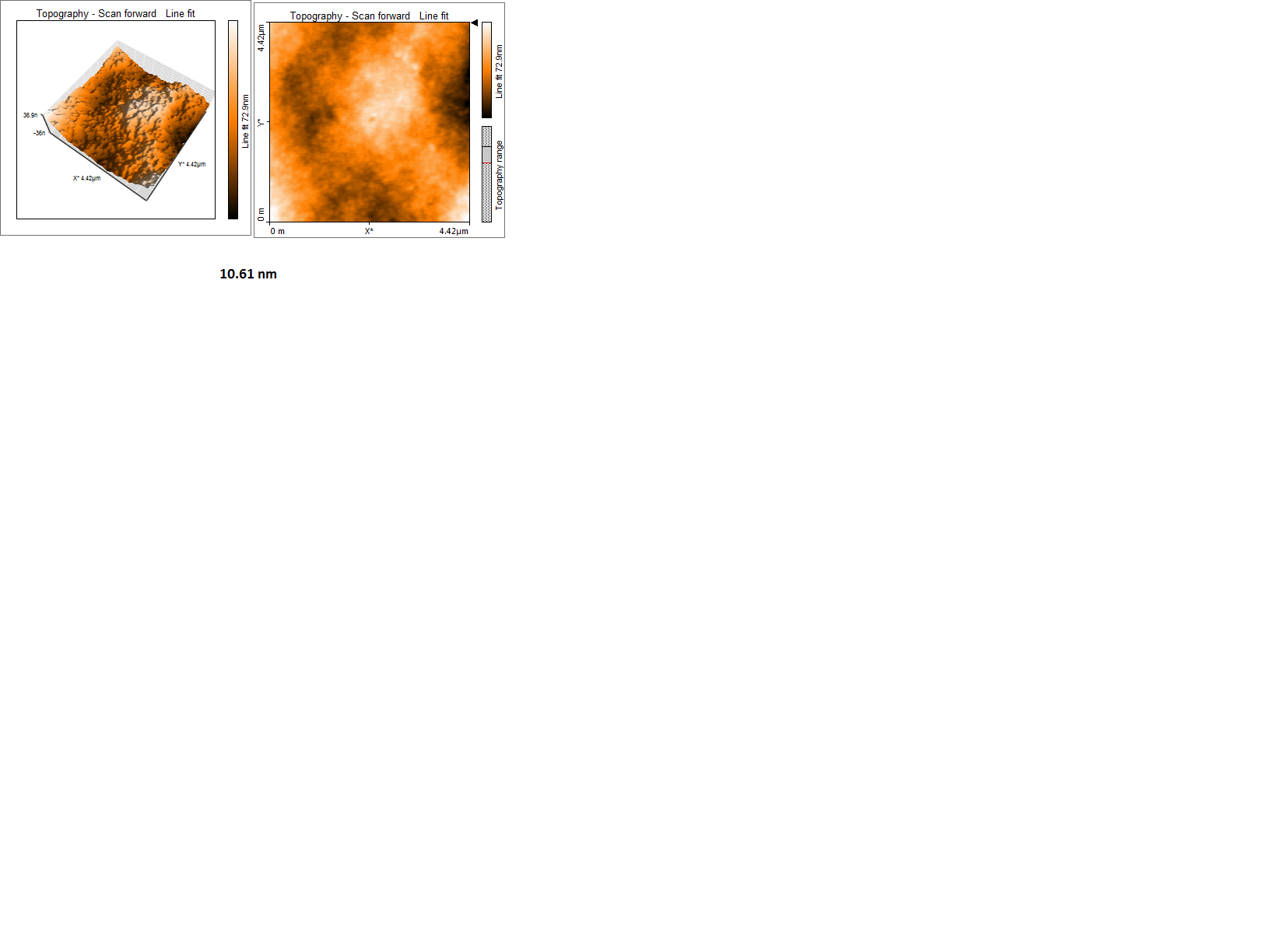 | 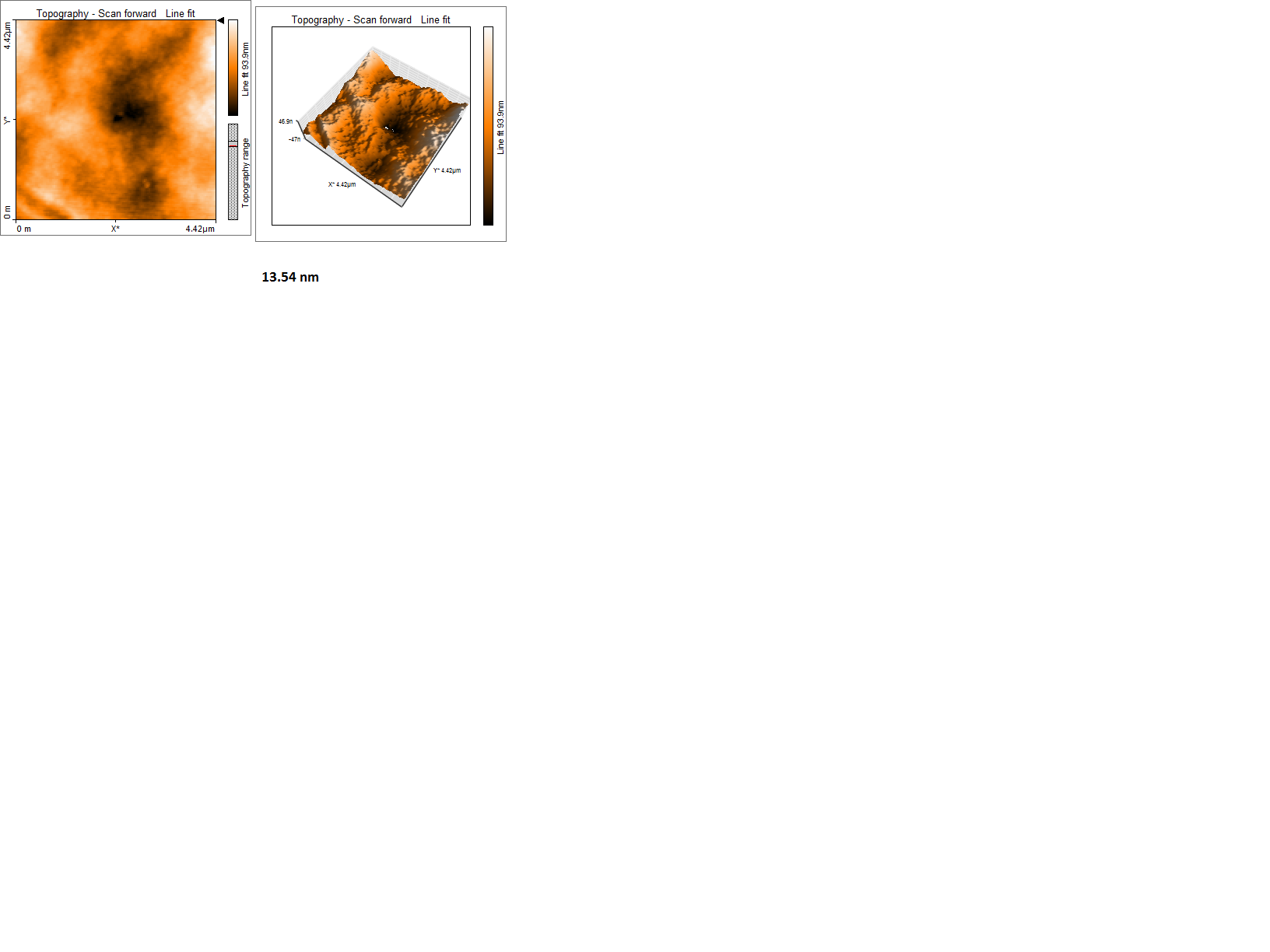 |
| M_3_ | M_4_ |
| 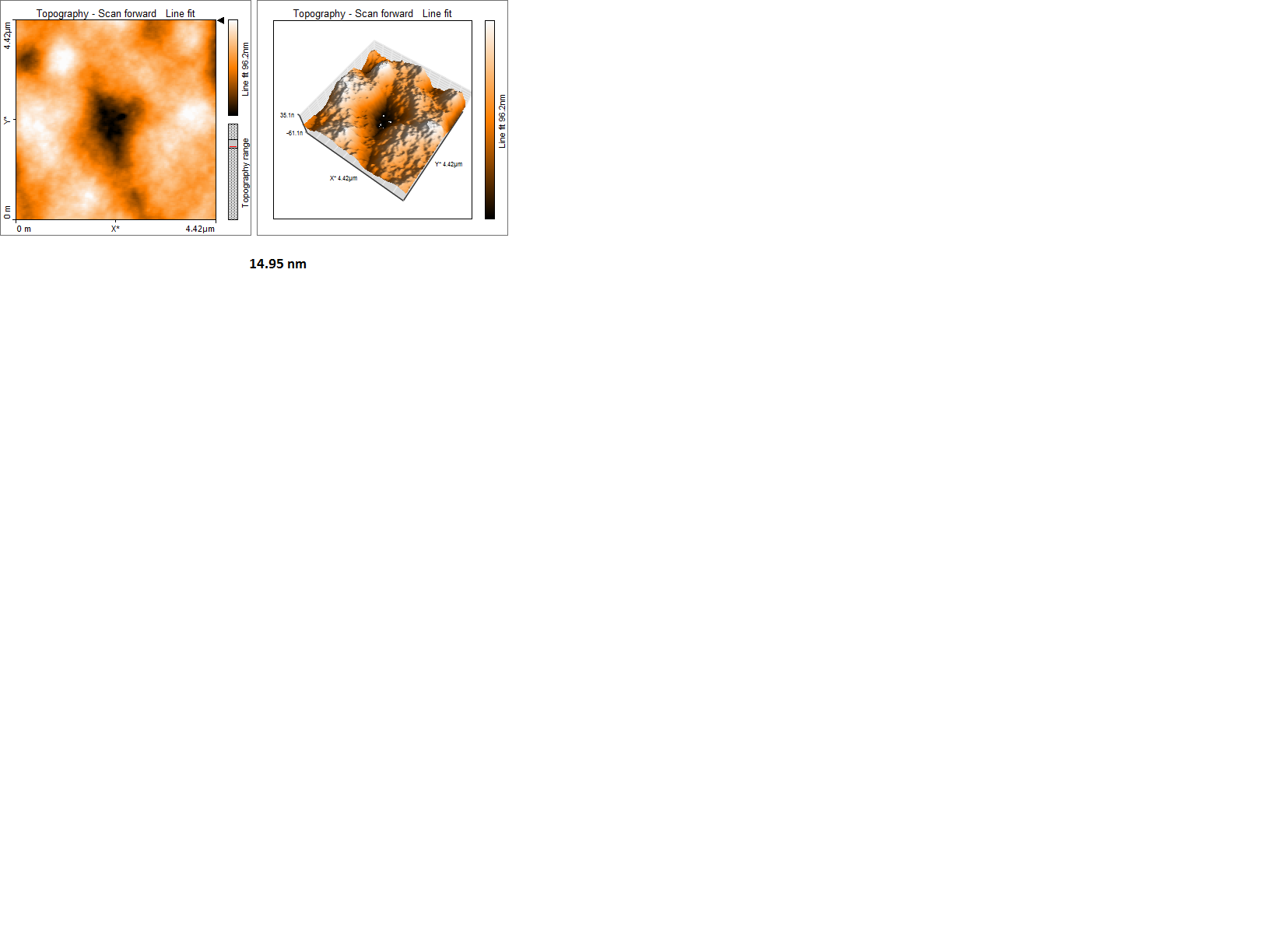 | 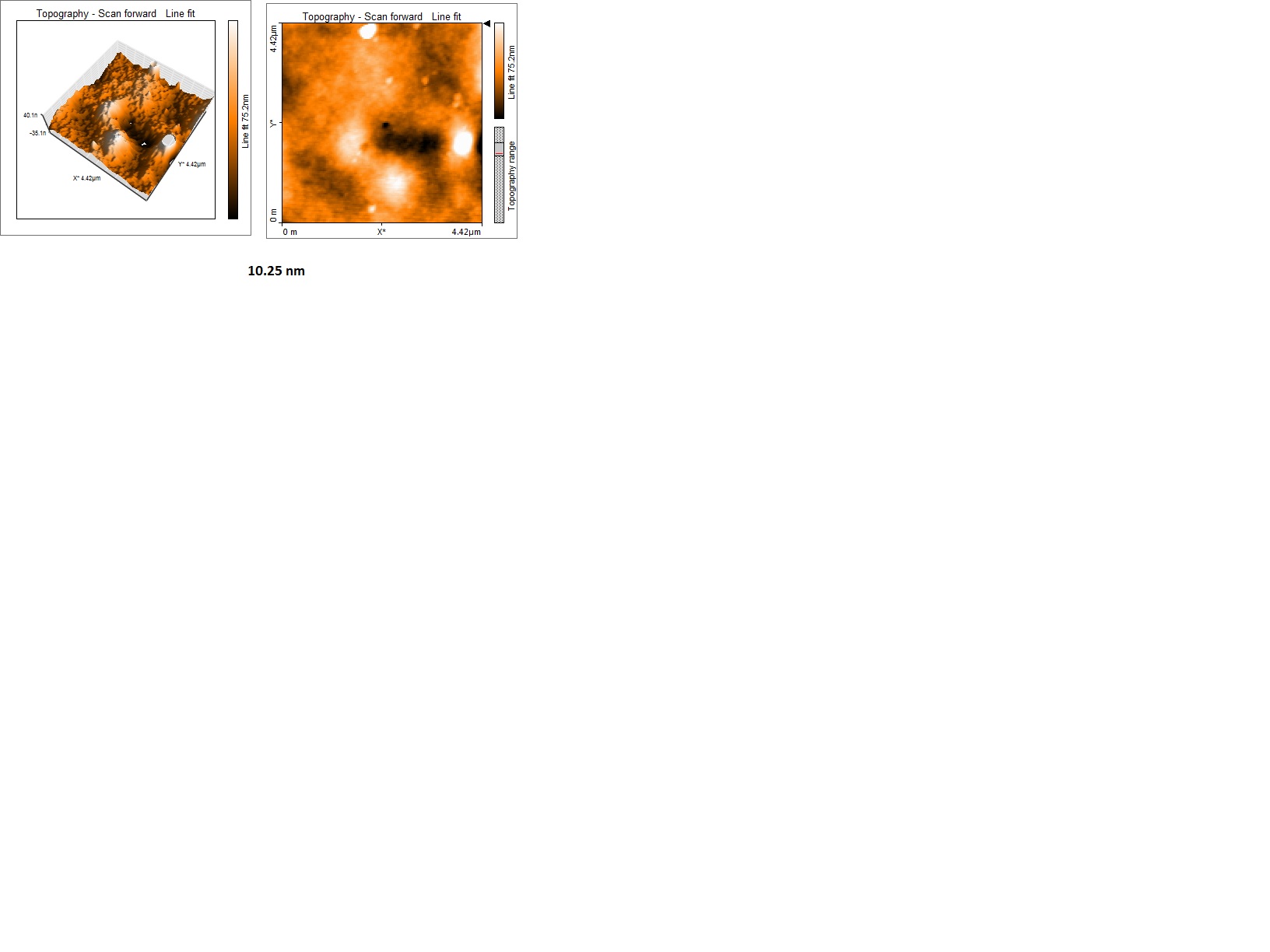 |
| M_5_ | M_6_ |
| 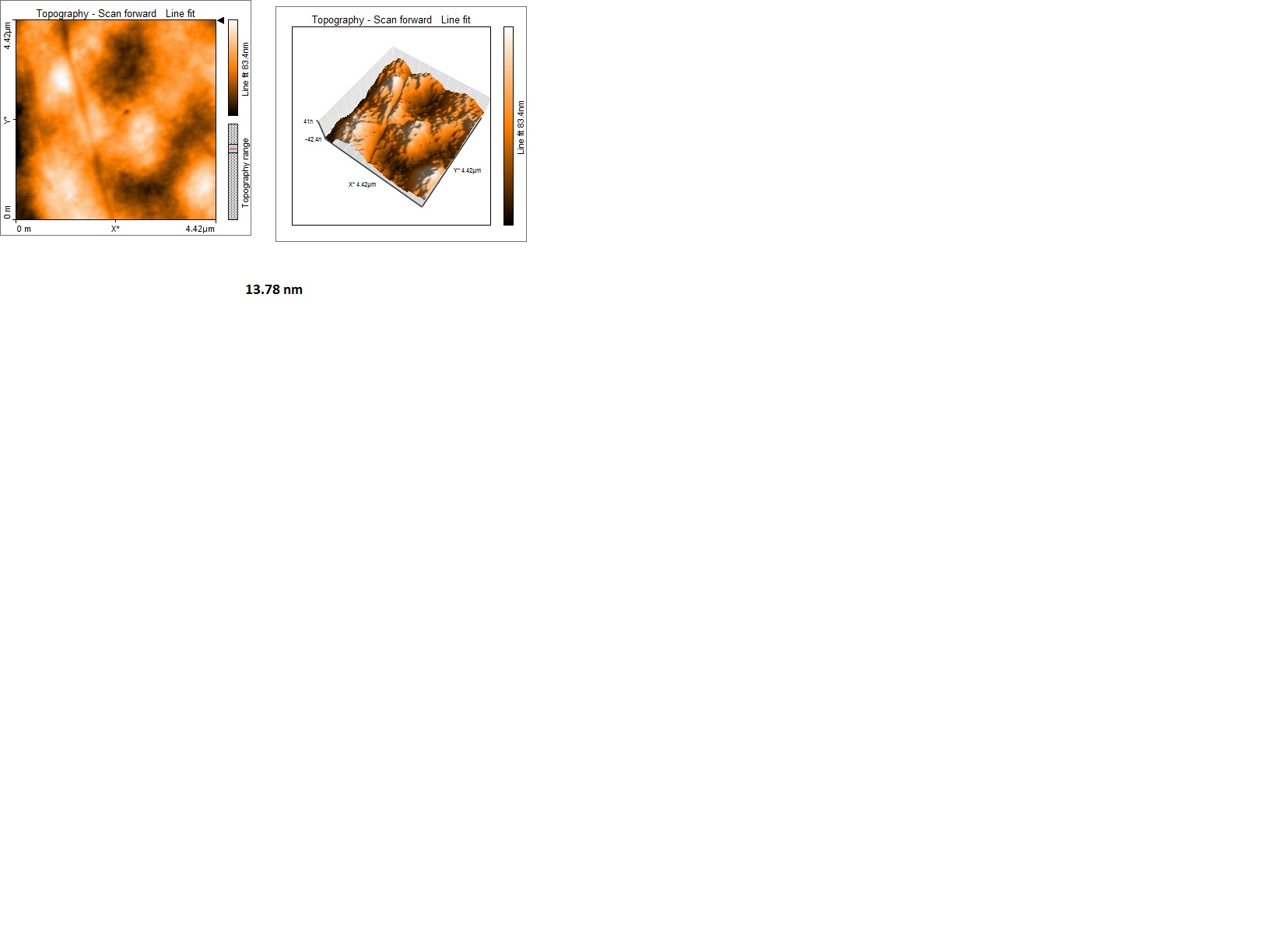 | 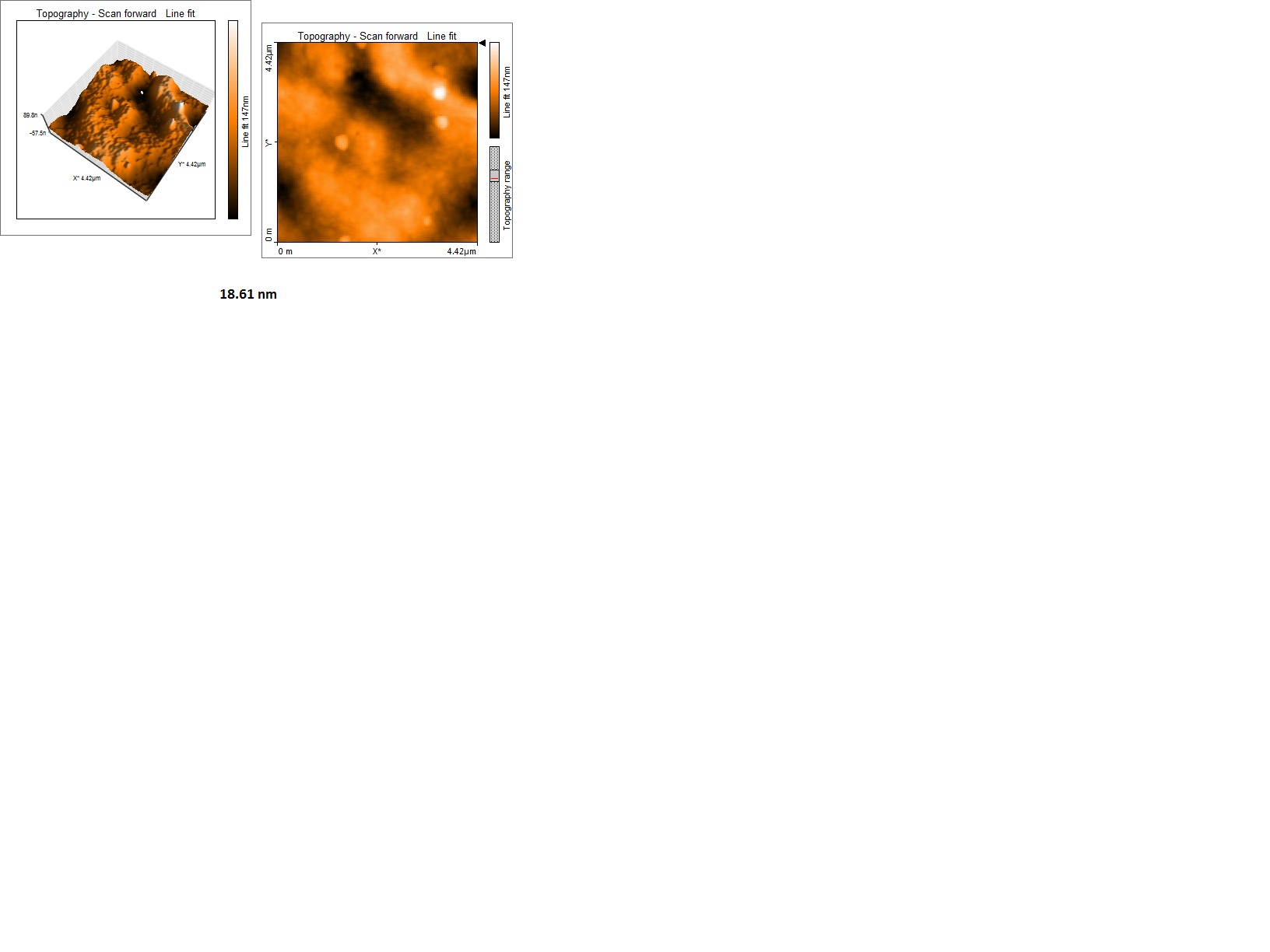 |
| M_7_ | M_8_ |
| 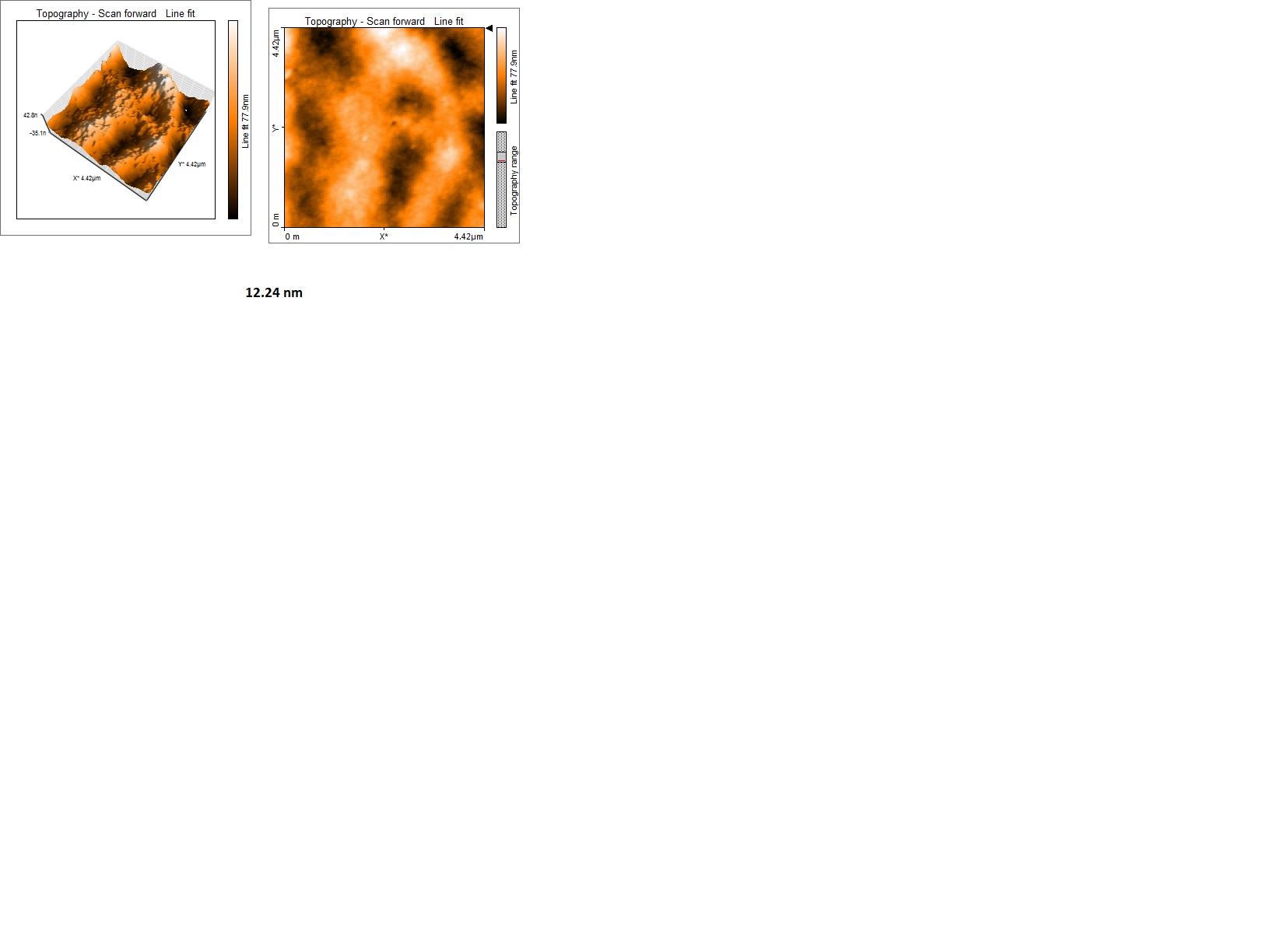 | 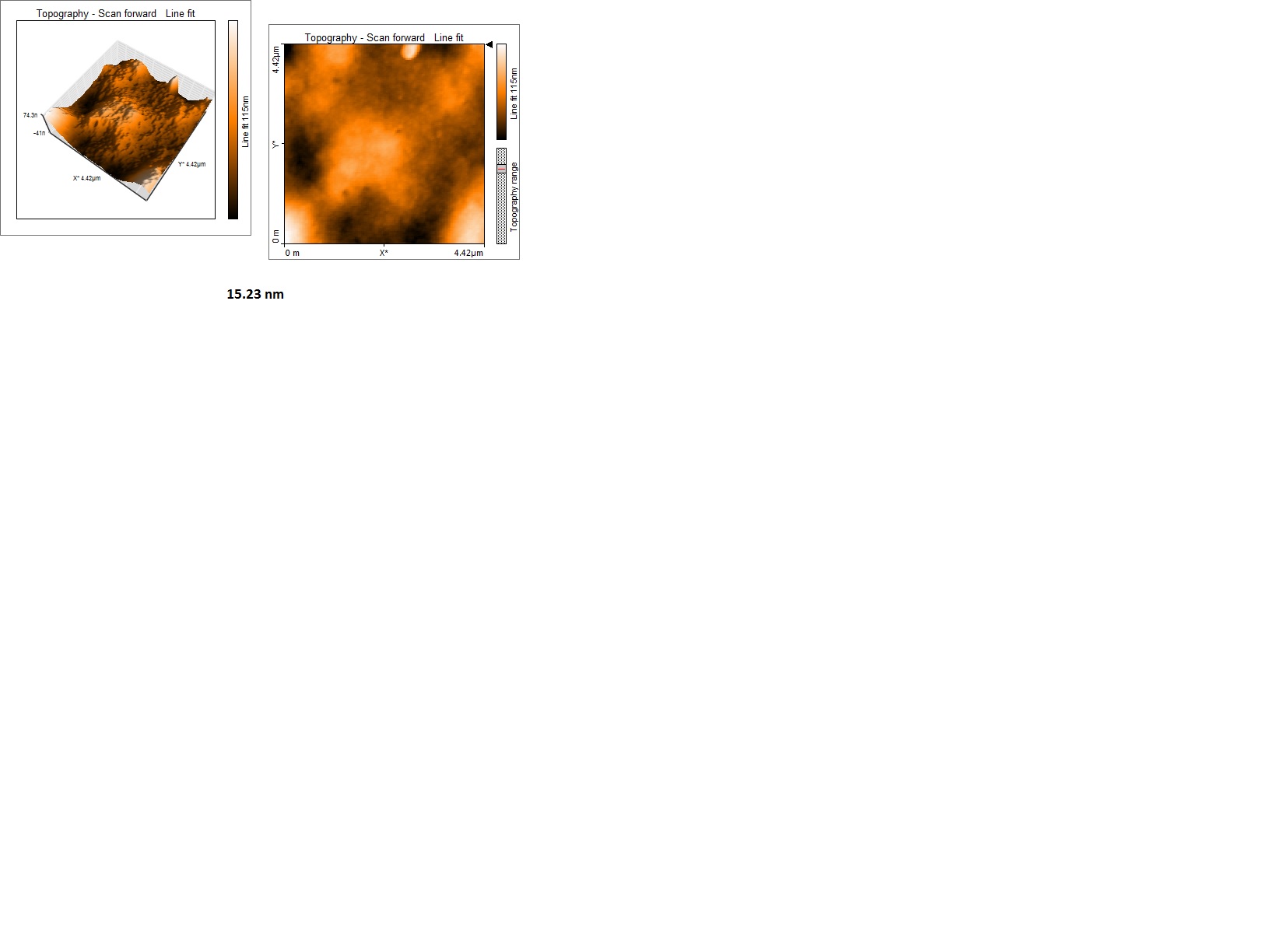 |
| M_9_ | M_10_ |
| Supplementary Fig. 2. 3D AFM images of DES-PEI membranes. | |
